# Plankton food webs in the oligotrophic Gulf of Mexico spawning grounds of Atlantic bluefin tuna

**DOI:** 10.1093/plankt/fbab023

**Published:** 2021-04-22

**Authors:** Michael R Stukel, Trika Gerard, Thomas B Kelly, Angela N Knapp, Raúl Laiz-Carrión, John T Lamkin, Michael R Landry, Estrella Malca, Karen E Selph, Akihiro Shiroza, Taylor A Shropshire, Rasmus Swalethorp

**Affiliations:** Department of Earth, Ocean and Atmospheric Science, Florida State University, Tallahassee, FL 32306, USA; Center for Ocean-Atmospheric Prediction Studies, Florida State University, Tallahassee, FL 32306, USA; Southeast Fisheries Science Center, National Marine Fisheries Service, National Oceanic and Atmospheric Administration (NOAA), Miami, FL 33149, USA; Department of Earth, Ocean and Atmospheric Science, Florida State University, Tallahassee, FL 32306, USA; Department of Earth, Ocean and Atmospheric Science, Florida State University, Tallahassee, FL 32306, USA; Centro Oceanográfico De Malaga, Instituto Español Del Oceanografía, Fuengirola, Spain; Southeast Fisheries Science Center, National Marine Fisheries Service, National Oceanic and Atmospheric Administration (NOAA), Miami, FL 33149, USA; Scripps Institution of Oceanography, University of California, San Diego, 9500 Gilman Dr., La Jolla, CA 92093-0227, USA; Cooperative Institute For Marine and Atmospheric Studies, University Of Miami, Miami, FL 33149, USA; Department of Oceanography, University of Hawaii At Manoa, Honolulu, HI 96822, USA; Cooperative Institute For Marine and Atmospheric Studies, University Of Miami, Miami, FL 33149, USA; Department of Earth, Ocean and Atmospheric Science, Florida State University, Tallahassee, FL 32306, USA; Center for Ocean-Atmospheric Prediction Studies, Florida State University, Tallahassee, FL 32306, USA; Scripps Institution of Oceanography, University of California, San Diego, 9500 Gilman Dr., La Jolla, CA 92093-0227, USA

**Keywords:** plankton ecology, marine food web, calanoid copepods, larval fish, nitrogen cycle

## Abstract

We used linear inverse ecosystem modeling techniques to assimilate data from extensive Lagrangian field experiments into a mass-balance constrained food web for the Gulf of Mexico open-ocean ecosystem. This region is highly oligotrophic, yet Atlantic bluefin tuna (ABT) travel long distances from feeding grounds in the North Atlantic to spawn there. Our results show extensive nutrient regeneration fueling primary productivity (mostly by cyanobacteria and other picophytoplankton) in the upper euphotic zone. The food web is dominated by the microbial loop (>70% of net primary productivity is respired by heterotrophic bacteria and protists that feed on them). By contrast, herbivorous food web pathways from phytoplankton to metazoan zooplankton process <10% of the net primary production in the mixed layer. Nevertheless, ABT larvae feed preferentially on podonid cladocerans and other suspension-feeding zooplankton, which in turn derive much of their nutrition from nano- and micro-phytoplankton (mixotrophic flagellates, and to a lesser extent, diatoms). This allows ABT larvae to maintain a comparatively low trophic level (~4.2 for preflexion and postflexion larvae), which increases trophic transfer from phytoplankton to larval fish.

## INTRODUCTION

The open-ocean Gulf of Mexico (GoM) is a nutrient-poor, low-plankton-biomass region (Biggs and Ressler, 2001; Muller-Karger *et al.*, 2015; Damien *et al.*, 2018; Shropshire *et al.*, 2020). Nevertheless, it is an important region for the spawning and larval development of many commercially important fishes (Lindo-Atichati *et al.*, 2012; Rooker *et al.*, 2012; Rooker *et al.*, 2013; Kitchens and Rooker, 2014; Cornic *et al.*, 2018). The western stock of Atlantic bluefin tuna (ABT) travel long distances from feeding grounds throughout the North Atlantic to spawning grounds in the oligotrophic GoM, implying that some characteristics of this region enhance larval success (Rooker *et al.*, 2007; Teo *et al.*, 2007; Rodríguez-Ezpeleta *et al.*, 2019). One strong possibility is that the low abundances of potential predators on eggs and larvae and the reduced competition for prey in this food-poor region are a prerequisite for pelagic larvae to survive to maturity (Biggs, 1992; Muhling *et al.*, 2017; Laiz-Carrión *et al.*, 2019; Shropshire *et al.*, this issue), but local enrichment processes such as fronts and eddies may also provide areas of higher productivity (Bakun, 2006), increasing the chances of larval survival (Bakun and Broad, 2003; Ciannelli *et al.*, 2015). ABT larvae could then exploit the available food resources which can transfer biomass originated from microbial loops to tuna larvae despite the low mean primary productivity. Nevertheless, it remains unclear how ABT and other GoM larval fishes manage to obtain sufficient nutrition during their critical first-feeding period. Discerning the structure of GoM planktonic food webs is crucial to answering such questions.

ABT larvae are selective feeders that rely disproportionately on specific prey taxa, including calanoid and poecilostomatoid copepods, cladocerans and appendicularians (Llopiz *et al.*, 2010; Llopiz *et al.*, 2015; Tilley *et al.*, 2016; Uriarte *et al.*, 2019; Shiroza *et al.*, this issue). These prey items, however, have distinctly different trophic and ecological roles (Landry *et al.*, 2019). Appendicularians are filter-feeding pelagic tunicates with fine meshes that give them access to some of the smallest cyanobacteria in the ocean (Alldredge, 1976; Gorsky and Fenaux, 1998). Poecilostomatoid copepods, by contrast, are predators of other metazoan zooplankton and hence likely feed comparatively high on the food chain (Turner, 1986). Cladocerans and calanoid copepods are often omnivorous filter feeders, although calanoid copepods can fill multiple trophic roles within the planktonic food web, including as predators on other metazoans (Uye and Kayano, 1994; Mauchline, 1998; Katechakis and Stibor, 2004; Bode *et al.*, 2015).

Elucidating the linkages between larval fish, their prey and the base of the planktonic food web is crucial to predicting climate change impacts on larval survival (Landry *et al.*, 2019). Different phytoplankton groups (e.g. *Prochlorococcus*, *Trichodesmium*, diatoms and mixotrophic nanoflagellates) will respond differently to warming, acidification and increased stratification in the oligotrophic ocean (Rost *et al.*, 2008; Flombaum *et al.*, 2013; Flynn *et al.*, 2013; Barton *et al.*, 2016; Hong *et al.*, 2017). These variable responses originate not only from different physiological responses to stressors but also due to the fundamentally different relationships between these groups and the limiting nutrient, light or temperature conditions. For instance: *Trichodesmium* and other diazotrophs (N_2_-fixing phytoplankton) are not nitrogen limited, *Prochlorococcus* is adapted to utilizing recycled nitrogen available at low concentrations in oligotrophic regions, and nanoflagellates may rely partially on phagotrophic behavior (mixotrophy) to alleviate nutrient stress (Scanlan and Post, 2008; Zehr, 2011; Stoecker *et al.*, 2017). The pathways that connect different nutrient sources (upwelling, lateral advection, recycled production and diazotrophy) through phytoplankton and zooplankton to larval fishes will determine how these organisms respond to climate change.

Here, we use linear inverse ecosystem models (LIEMs) as a data synthesis tool to constrain pelagic food webs of the oligotrophic GoM. We utilize results from field experiments designed to investigate the open-ocean GoM ecosystem from nutrients to fish (Gerard *et al.*, this issue). LIEM allows us to incorporate diverse ecosystem measurements (e.g. primary productivity, protistan grazing rates, copepod δ^15^N and larval ABT gut contents) into a mass-balance constrained ecosystem model. We use the results to address four distinct questions: What is the trophic level (TL) of larval ABT? What is the trophic efficiency of food chains leading to larval ABT? Which phytoplankton groups ultimately support secondary production by larval ABT? What nitrogen sources support the specific food web pathways utilized by larval ABT?

**Table I:** Rate, biomass and δ^15^N measurements used as inputs to the inverse model

	Units	C1	C5	Source
Rate measurements				
NPP (shallow)	mmol N m^−2^ d^−1^	2.23 ± 0.13	3.08 ± 0.14	Yingling *et al.* (this issue)
NPP (deep)	mmol N m^−2^ d^−1^	1.64 ± 0.07	1.34 ± 0.05	Yingling *et al.* (this issue)
*f*-ratio (shallow)	mmol N m^−2^ d^−1^	0.06 ± 0.04	0.14 ± 0.04	Yingling *et al.* (this issue)
*f*-ratio (deep)	mmol N m^−2^ d^−1^	0.44 ± 0.3	0.09 ± 0.02	Yingling *et al.* (this issue)
Protistan grazing rate (shallow)	mmol N m^−2^ d^−1^	1.72 ± 0.6	2.58 ± 0.14	Yingling *et al.* (this issue)
Protistan grazing rate (deep)	mmol N m^−2^ d^−1^	1.44 ± 0.48	0.66 ± 0.18	Yingling *et al.* (this issue)
Picophyto NPP (shallow)	mmol N m^−2^ d^−1^	1.15 ± 0.21	2.38 ± 0.37	Landry *et al.* (this issue)
Picophyto NPP (deep)	mmol N m^−2^ d^−1^	0.78 ± 0.15	1.02 ± 0.17	Landry *et al.* (this issue)
Flagellate NPP (shallow)	mmol N m^−2^ d^−1^	1.01 ± 0.37	0.69 ± 0.17	Landry *et al.* (this issue)
Flagellate NPP (deep)	mmol N m^−2^ d^−1^	0.83 ± 0.35	0.32 ± 0.13	Landry *et al.* (this issue)
Diatom NPP (shallow)	mmol N m^−2^ d^−1^	0.08 ± 0.03	0.01 ± 0	Landry *et al.* (this issue)
Diatom NPP (deep)	mmol N m^−2^ d^−1^	0.02 ± 0.02	0 ± 0	Landry *et al.* (this issue)
Picophyto mortality (shallow)	mmol N m^−2^ d^−1^	0.71 ± 0.32	1.6 ± 0.12	Landry *et al.* (this issue)
Picophyto mortality (deep)	mmol N m^−2^ d^−1^	0.43 ± 0.07	0.34 ± 0.09	Landry *et al.* (this issue)
Flagellate mortality (shallow)	mmol N m^−2^ d^−1^	0.86 ± 0.26	0.19 ± 0.11	Landry *et al.* (this issue)
Flagellate mortality (deep)	mmol N m^−2^ d^−1^	0.6 ± 0.17	0.04 ± 0.02	Landry *et al.* (this issue)
Diatom mortality (shallow)	mmol N m^−2^ d^−1^	0.04 ± 0.02	0.01 ± 0	Landry *et al.* (this issue)
Diatom mortality (deep)	mmol N m^−2^ d^−1^	0.01 ± 0.01	0 ± 0	Landry *et al.* (this issue)
NVM mesozoo grazing	mmol N m^−2^ d^−1^	0.39 ± 0.1	0.52 ± 0.1	Landry and Swalethorp (this issue)
VM mesozoo grazing	mmol N m^−2^ d^−1^	0.03 ± 0.06	0.15 ± 0.08	Landry and Swalethorp (this issue)
SedTrap flux (shallow)	mmol N m^−2^ d^−1^	1.53 ± 0.55	1.08 ± 0.07	Stukel *et al.* (this issue)
SedTrap flux (deep)	mmol N m^−2^ d^−1^	0.46 ± 0.02	0.87 ± 0.18	Stukel *et al.* (this issue)
Chl sinking (shallow)	mmol N m^−2^ d^−1^	0.02 ± 0.02	0.03 ± 0.02	Stukel *et al.* (this issue)
Chl sinking (deep)	mmol N m^−2^ d^−1^	0.02 ± 0.01	0.05 ± 0.04	Stukel *et al.* (this issue)
Fecal pellet sinking (shallow)	mmol N m^−2^ d^−1^	0.03 ± 0.03	0.02 ± 0.02	Stukel *et al.* (this issue)
Fecal pellet sinking (deep)	mmol N m^−2^ d^−1^	0.13 ± 0.06	0.25 ± 0.2	Stukel *et al.* (this issue)
Microzoo to preflex	nmol N m^−2^ d^−1^	0.34 ± 0.16	0 ± 0.01	Shiroza *et al.* (this issue)
Microzoo to postflex	nmol N m^−2^ d^−1^	5.45 ± 0.88	0 ± 0.01	Shiroza *et al.* (this issue)
Appendicularian to preflex	nmol N m^−2^ d^−1^	0.77 ± 0.32	0.04 ± 0.03	Shiroza *et al.* (this issue)
Appendicularian to postflex	nmol N m^−2^ d^−1^	6.75 ± 0.47	1.88 ± 0.2	Shiroza *et al.* (this issue)
Cladoceran to preflex	nmol N m^−2^ d^−1^	0.16 ± 0.16	0.78 ± 0.25	Shiroza *et al.* (this issue)
Cladoceran to postflex	nmol N m^−2^ d^−1^	23.43 ± 2.26	26.47 ± 2.78	Shiroza *et al.* (this issue)
Calanoids to preflex	nmol N m^−2^ d^−1^	3.61 ± 1.34	1.87 ± 0.35	Shiroza *et al.* (this issue)
Calanoids to postflex	nmol N m^−2^ d^−1^	63.62 ± 0.96	13.49 ± 0.32	Shiroza *et al.* (this issue)
Poecilastomatoids to preflex	nmol N m^−2^ d^−1^	0 ± 0.01	0 ± 0.01	Shiroza *et al.* (this issue)
Poecilastomatoids to postflex	nmol N m^−2^ d^−1^	1.74 ± 2.63	0.89 ± 0.03	Shiroza *et al.* (this issue)
Biomass and other measurements				
Temperature (0–50)	°C	24.31	24.44	CTD
Temperature (50–120)	°C	22.14	21.68	CTD
Temperature (120–300)	°C	16.41	16.46	CTD
HerbNVM biomass (shallow)	μmol N m^−2^	23.24	45.63	Shiroza *et al.* (this issue)
App biomass (shallow)	μmol N m^−2^	0.22	0.32	Shiroza *et al.* (this issue)
Clad biomass (shallow)	μmol N m^−2^	0.06	0.12	Shiroza *et al.* (this issue)
NVM Cal biomass (shallow)	μmol N m^−2^	103.09	126.89	Shiroza *et al.* (this issue)
Chaeto biomass (shallow)	μmol N m^−2^	107.04	130.13	Shiroza *et al.* (this issue)
Poecil biomass (shallow)	μmol N m^−2^	28.65	18.53	Shiroza *et al.* (this issue)
Preflex biomass (shallow)	μmol N m^−2^	0.06	1.18	Shiroza *et al.* (this issue)
Postflex biomass (shallow)	μmol N m^−2^	0.62	1.91	Shiroza *et al.* (this issue)
HerbVM biomass	μmol N m^−2^	88.62		Shiroza *et al.* (this issue)
vmCal biomass	μmol N m^−2^	78.29		Shiroza *et al.* (this issue)
Cyano biomass (shallow)	mmol N m^−2^	9.39	18.39	Selph *et al.* (this issue)
Tricho biomass (shallow)	μmol N m^−2^	27.23	0.97	Selph *et al.* (this issue)
Diatom biomass (shallow)	mmol N m^−2^	0.13	0.08	Selph *et al.* (this issue)
Flag biomass (shallow)	mmol N m^−2^	4.84	2.74	Selph *et al.* (this issue)
Cyano biomass (deep)	mmol N m^−2^	8.22	6.77	Selph *et al.* (this issue)
Tricho biomass (deep)	μmol N m^−2^	0.77	0.11	Selph *et al.* (this issue)
Diatom biomass (deep)	mmol N m^−2^	0.12	0.04	Selph *et al.* (this issue)
Flag biomass (deep)	mmol N m^−2^	6.97	3.63	Selph *et al.* (this issue)
HerbNVM size	μg C ind^−1^	1.35	1.35	Shiroza *et al.* (this issue)
App size	μg C ind^−1^	0.07	0.07	Shiroza *et al.* (this issue)
Clad size	μg C ind^−1^	0.68	0.68	Shiroza *et al.* (this issue)
NVM Cal size	μg C ind^−1^	4.44	4.44	Shiroza *et al.* (this issue)
Chaeto size	μg C ind^−1^	20.83	20.83	Shiroza *et al.* (this issue)
Poecil size	μg C ind^−1^	5.33	5.33	Shiroza *et al.* (this issue)
Preflex size	μg C ind^−1^	83.71	83.71	Shiroza *et al.* (this issue)
Postflex size	μg C ind^−1^	179.75	179.75	Shiroza *et al.* (this issue)
HerbVM size	μg C ind^−1^	4.44	4.44	Shiroza *et al.* (this issue)
vmCal size	μg C ind^−1^	4.44	4.44	Shiroza *et al.* (this issue)
Maximum upwelling rate (shallow)	μmol N m^−2^ d^−1^	0.09	0.09	Kelly *et al.* (in review)
Maximum upwelling rate (deep)	μmol N m^−2^ d^−1^	366.79	1543.33	Kelly *et al.* (in review)
Maximum lateral advection of PON	mmol N m^−2^ d^−1^	3.22	3.22	Kelly *et al.* (in review)
Maximum lateral advection of DON	mmol N m^−2^ d^−1^	1.56	1.56	Kelly *et al.* (in review)
δ^15^N values				
Upwelled nitrate	δ^15^N_AIR_ (‰)	3.20	2.90	Knapp *et al.* (this issue)
Preflex ABT	δ^15^N_AIR_ (‰)	4.63	7.50	Swalethorp *et al.* (unpub.)
Postflex ABT	δ^15^N_AIR_ (‰)	4.21	6.16	Swalethorp *et al.* (unpub.)
Shallow SedTrap	δ^15^N_AIR_ (‰)	2.90	3.80	Stukel *et al.* (this issue)
Deep SedTrap	δ^15^N_AIR_ (‰)	4.89	4.55	Stukel *et al.* (this issue)
Shallow DON	δ^15^N_AIR_ (‰)	3.37	3.27	Knapp *et al.* (this issue)
Deep DON	δ^15^N_AIR_ (‰)	3.31	3.39	Knapp *et al.* (this issue)
Shallow PON	δ^15^N_AIR_ (‰)	1.44	2.66	Stukel *et al.* (this issue)
Deep PON	δ^15^N_AIR_ (‰)	1.80	1.63	Stukel *et al.* (this issue)
Appendicularian	δ^15^N_AIR_ (‰)	2.42	5.12	Swalethorp *et al.* (unpub).
Calanoid copepods	δ^15^N_AIR_ (‰)	3.12	4.67	Swalethorp *et al.* (unpub).
Chaetognaths	δ^15^N_AIR_ (‰)	5.70	7.58	Swalethorp *et al.* (unpub).
HerbVM	δ^15^N_AIR_ (‰)	4.73	5.88	Swalethorp *et al.* (unpub).
HerbNVM	δ^15^N_AIR_ (‰)	3.22	3.98	Swalethorp *et al.* (unpub).
Poecilostomatoids	δ^15^N_AIR_ (‰)		6.29	Swalethorp *et al.* (unpub).
Cladocerans	δ^15^N_AIR_ (‰)	1.48	5.16	Swalethorp *et al.* (unpub).

## METHODS

### *In situ* measurements

Our data are derived from two cruises in ABT spawning grounds in April–May 2017 and 2018 as part of the Bluefin Larvae in Oligotrophic Ocean Foodwebs: Investigating Nutrients to Zooplankton in the Gulf of Mexico (BLOOFINZ–GoM) Project (Table I). During these cruises, we conducted regional zooplankton sampling surveys, which were guided partly by the Bluefin Tuna Index (Domingues *et al.*, 2016), to identify contrasting open-ocean water parcels with and without high abundances of ABT larvae (Gerard *et al.*, this issue). We then conducted 3- to 5-day Lagrangian experiments (hereafter ‘cycles’), while following satellite-enabled drift arrays with 3- × 1-m holey-sock drogues centered at 15 m depth, which allowed us to follow patches of mixed-layer water (Landry *et al.*, 2009; Stukel *et al.*, 2015). Five experimental cycles were conducted; in this study, however, we focus only on two experimental cycles with high larval ABT abundance—hereafter, Cycle 1 (C1) from the 2017 cruise and Cycle 5 (C5) from the 2018 cruise.

During each cycle, we conducted daily profiles with a CTD-Niskin rosette to measure temperature, salinity and density and to collect samples for chlorophyll *a* measurements (acidification method; Strickland and Parsons, 1972), phytoplankton pigment analyses (high-pressure liquid chromatography), picophytoplankton and heterotrophic bacteria enumeration (by flow cytometry; Selph *et al.*, 2016; Selph *et al.*, this issue), nano- and micro-phytoplankton biomass (Taylor and Landry, 2018; Selph *et al.*, this issue), *Trichodemium* biomass (Selph *et al.*, this issue), nutrients (nitrate and ammonium; Knapp *et al.*, this issue), dissolved organic nitrogen (DON; Knapp *et al.*, this issue), particulate organic nitrogen (PON; Stukel *et al.*, this issue) and δ^15^N of nitrate, DON and PON (Knapp *et al.*, this issue; Stukel *et al.*, this issue).

We also conducted a suite of daily *in situ* rate measurements that were incubated in mesh bags affixed at six depths spanning the euphotic zone on one of the floating arrays. These measurements included nitrate uptake (Yingling *et al.*, this issue), net primary production (NPP) (Yingling *et al.*, this issue) and group-specific phytoplankton growth and mortality due to protistan grazing (Landry *et al.*, 2016; Landry *et al.*, this issue). All *in situ* incubations were conducted for 24 h at natural light and temperature conditions. We also conducted shorter (4–6 h) shipboard incubations for nitrate and ammonium uptake (Yingling *et al.*, this issue).

Twice per day (midday and midnight), we conducted oblique net tows through the euphotic zone to collect mesozooplankton that were analyzed for carbon, nitrogen, isotopes and gut pigment content (Landry and Swalethorp, this issue). Gut pigment contents were analyzed as in Décima *et al.* (2016) to estimate grazing rates (Landry and Swalethorp, this issue). ABT larvae were sampled frequently by standard double oblique tows (~8 tows d^−1^) with a 90-cm square bongo net (500-μm mesh) mounted with flowmeters to a depth of 25 m to ensure that we remained inside ABT habitat. Individual tuna larvae (2055 larvae, ranging from 3 to 9 mm length) were sorted onboard, and the identified ABT were liquid nitrogen- or ethanol-preserved for further analysis of abundance, body size, dry weight, gut content, otolith-based age and isotopic measurements (Laiz-Carrion *et al.*, 2015; García *et al.*, 2017; Malca *et al.*, 2017; Laiz-Carrión *et al.*, 2019; Malca *et al.*, in prep.; Shiroza *et al.*, this issue).

Nitrogen inputs to and outputs from the euphotic zone were constrained using sediment traps, Thorpe-scale analyses and remote-sensing products of lateral PON transport. Surface-tethered drifting sediment traps were used to collect sinking PON, chlorophyll and phaeopigments at 50 m depth, near the base of the euphotic zone (~120 m), and beneath the euphotic zone (200 m) (Stukel *et al.*, this issue). We used Thorpe-scale analyses and nitrate concentration profiles to constrain vertical eddy diffusivity and upward nitrate flux (Gargett and Garner, 2008; Kelly *et al.*, in review). We combined day–night differences in mesozooplankton biomass with allometric ammonium–excretion relationships to quantify active transport by diel vertical migrants (Ikeda, 1985; Landry and Swalethorp, this issue). We also quantified lateral transport of organic matter into the oligotrophic GoM using two independent approaches: a combination of remote-sensing-derived estimates of currents with remote-sensing-derived particulate carbon and a biogeochemical model developed for the open-ocean GoM (Shropshire *et al.*, 2020; Kelly *et al.*, in review).

### Food web structure

**Fig. 1:**
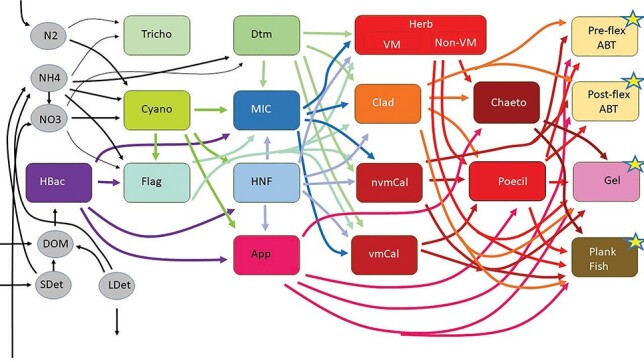
Food web structure. All major food web flows between living organism groups are shown. However, for visual simplicity, we omit production of NH_4_^+^, DOM and detritus by all living groups as well as consumption of detritus by protistan zooplankton and suspension-feeding metazoans. Stars indicate groups at the highest TLs, for which secondary production is a model closure term. The model has a two-layer structure (~mixed layer and deep euphotic zone) with all trophic components in both layers, except for larval ABT. For all model flows, see Supplementary Table SI (see online supplementary data at *Journal of Plankton Research* online). HBac, heterotrophic bacteria; SDet, small detritus; LDet, large (sinking) detritus; Tricho, *Trichodesmium*; Cyano, cyanobacteria; Flag, mixotrophic flagellates; Dtm, diatoms; MIC, microzooplankton; HNF, heterotrophic nanoflagellates; App, appendicularians; HerbVM, vertically migrating herbivorous suspension feeders; HerbNVM, non-vertically migrating herbivorous suspension feeders; Clad, cladocerans; nvmCal, non-vertically migrating calanoid copepods; vmCal, vertically migrating calanoid copepods; Chaeto, chaetognaths; Poecil, poecilostomatoid copepods; Preflex ABT, preflexion ABT; Postflex ABT, postflexion ABT; Gel, gelatinous predators (ctenophores and cnidarians); Plank Fish, planktivorous fish.

Our food web structure was specifically designed to address the variability in trophic pathways within GoM foodwebs that channel energy toward the prey of ABT larvae (either efficiently or inefficiently) or to the multiple plankton taxa that are not suitable prey for the ABT larvae (Fig. 1). The model includes three inorganic N classes (NO_3_^−^, NH_4_^+^ and N_2_) and three non-living organic matter pools [dissolved organic matter (DOM), small detritus and large detritus]. It includes four phytoplankton: *Trichodesmium*, picophytoplankton (assumed to be potentially diazotrophic), diatoms and mixotrophic flagellates. It also includes heterotrophic bacteria, heterotrophic nanoflagellates and microzooplankton. Six suspension-feeding mesozooplankton are included: appendicularians (the only suspension feeders capable of feeding on cyanobacteria and heterotrophic bacteria), vertically migrating calanoid copepods, non-vertically migrating calanoid copepods, cladocerans, other non-vertically migrating herbivorous suspension feeders and other vertically migrating herbivorous suspension feeders. It includes two small predatory mesozooplankton: chaetognaths and poecilostomatoid copepods. It also includes four ‘higher TLs’ that serve as closure terms in the model: preflexion ABT larvae, postflexion ABT larvae, other planktivorous fish and predatory gelatinous zooplankton (e.g. ctenophores and cnidarians). ABT are assumed to feed on microzooplankton, appendicularians, cladocerans, non-vertically migrating calanoid copepods and poecilostomatoid copepods. Piscivory is not included in the model because field results showed that ichthyoplankton are not important prey to the 3–9-mm larvae (Shiroza *et al.*, this issue). However, piscivory should be added if the model is used for larger larvae or in regions with higher ichthyoplankton densities. Other trophic pathways are determined based on known predator–prey relationships. Because ABT larvae feed only in the mixed layer, we include two layers in the model: upper euphotic zone (0–50 m) and deep euphotic zone (50–100 m on C1; 50–85 m on C5). All model compartments are identical, except that ABT larvae only exist in the upper euphotic zone. The two layers are connected through upward flux of nitrate, downward flux of sinking particles and the motions of vertical-migratory taxa, which are assumed to freely migrate into and between the two layers during the night, but reside beneath the euphotic zone (i.e. outside the model) during the day. Inputs to the model include upwelled nitrate, diazotrophy and lateral advection of particulate organic matter (POM) and DOM. Closure terms include secondary production of higher TLs, sinking of large detritus, sinking of diatoms, sinking of mixotrophic flagellates and excretion from vertical migratory taxa beneath the euphotic zone. We assume Redfield stoichiometry for all model flows, which allows us to relate respiration to ammonium excretion. We thus use the term ‘respiration’ when relating respiratory or excretory fluxes to primary production and the term ‘excretion’ when discussing nutrient recycling. Supplementary Table SI (see online supplementary data at *Journal of Plankton Research* online) shows all model flows.

### Inverse model solution

To constrain the flux of nitrogen through unmeasured ecosystem pathways, we used LIEM techniques (Vézina and Platt, 1988; van Oevelen *et al.*, 2010) to specify mass-balance constraints that must be exactly fit by food-web solutions, approximate equations that quantify measured rates with associated measurement uncertainty and inequality constraints that represent *a priori* acceptable ranges for different ecosystem properties (e.g. gross growth efficiency varies from 10 to 40%). We used a total of 44 mass-balance constraints, 80 approximate equalities and 533 inequality constraints. However, with 302 total unknown food-web flows, the system remains under-constrained. To objectively determine representative solutions (and confidence limits) for all flows, we used the Markov Chain Monte Carlo (MCMC) with ^15^N approach (Stukel *et al.*, 2018a, b). The MCMC approach conducts a random walk through the solution space that is constrained to fit the exact equations and bounded by the inequality constraints (Kones *et al.*, 2009; Soetaert *et al.*, 2009; Van den Meersche *et al.*, 2009). New solutions are accepted based on the relative misfits of the new and previous solutions with respect to the approximate equality measurements. The mean solution of the MCMC approach has been shown to more accurately recover withheld measurement constraints than the previously used L_2_ minimum norm approach (Stukel *et al.*, 2012; Saint-Béat *et al.*, 2013). The MCMC+^15^N approach used herein allows for the incorporation of non-linear constraints associated with unknown δ^15^N values for some organisms or non-living nitrogen pools in the ecosystem to further constrain the system. For additional details, see the online supplementary appendix (see online supplementary data at *Journal of Plankton Research* online).

### Food web analyses

TLs for all zooplankton were computed as one plus the ingestion-weighted mean TL of prey (${\mathrm{TL}}_{\mathrm{consumer}}=\sum ({\mathrm{TL}}_{\mathrm{prey},i}\times{F}_{\mathrm{prey},i\to \mathrm{consumer}})/\sum{F}_{\mathrm{prey},i\to \mathrm{consumer}}$, where ${\mathrm{TL}}_{\mathrm{prey},i}$ is the TL of prey *i*, and ${F}_{\mathrm{prey},i\to \mathrm{consumer}}$ is the rate of feeding of the consumer on prey *i*. All phytoplankton were assumed to have a TL = 1, except mixotrophic flagellates, which had $\mathrm{TL}=(1-{p}_{\mathrm{phag}})+{p}_{\mathrm{phag}}(1+{\mathrm{TL}}_{\mathrm{prey}})$, where *p*_phag_ is the proportion of their nitrogen derived from phagotrophy (rather than dissolved nutrient uptake). Heterotrophic bacteria were assumed to have a TL equal to 1 plus the TL of the organism producing the organic matter they utilized.

To quantify indirect nitrogen flows through the food web, we used indirect food web flow analysis (Hannon, 1973). The normalized amount of nitrogen (direct and indirect) that any organism derives from any other organism (or non-living nitrogen pool) can be computed as ${(I-G)}^{-1}$, where *I* is the identity matrix and *G* is the normalized production matrix (i.e. a matrix giving the percentage of an organism’s nitrogen requirement derived from any other organism).

Following Stukel *et al*. (2012), we defined three major food web pathways that describe energy and nutrient fluxes from the base of the food web: the herbivorous food chain, the multivorous food chain and the microbial loop. (i) The herbivorous food chain = the sum of direct nitrogen flux from phytoplankton to metazoan zooplankton. (ii) The multivorous food chain = the sum of nitrogen flux that reaches metazoan zooplankton after passing through protistan grazers. (iii) The microbial loop = the sum of bacterial respiration and the fraction of protistan respiration that was supported by bacterial production. Results for each parameter are presented as means and 95% confidence intervals (CIs).

## RESULTS

### Model performance

**Fig. 2:**
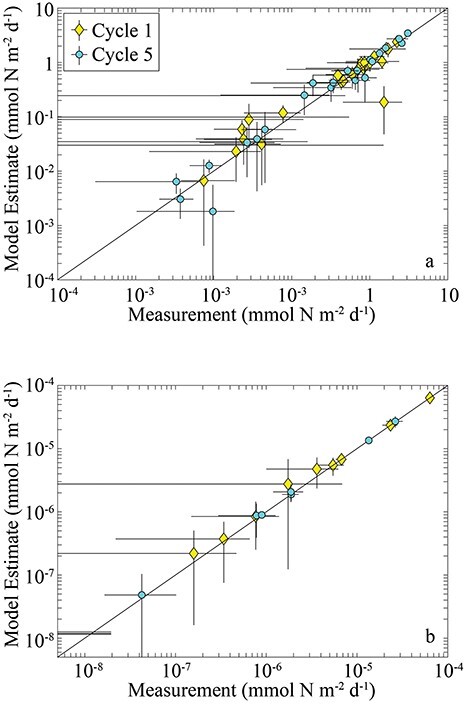
Comparison between field measurements and model estimates for planktonic ecosystem rates (**a**) and ABT feeding measurements (**b**). Yellow diamonds are C1, cyan circles are C5.

The LIEM demonstrates close agreement with field measurements. The square root mean squared error (SRMSE), which can be thought of as the average number of standard errors that model estimates were from the measurements, was 1.17 for C1 if we consider only the field rate measurements and 1.53 for all approximate equality equations (including the δ^15^N mass-balance equations). For C5, the equivalent values were 1.40 and 1.65. One of the largest model-data mismatches was for sinking flux from the shallow to the deep euphotic zone during C1. The model struggled to find solutions that matched observations showing 3-fold higher sinking nitrogen flux from the upper euphotic zone to the lower euphotic zone than out of the euphotic zone. The model also slightly overestimated the grazing of suspension-feeding zooplankton on phytoplankton during both cycles, although in this case, the model’s 95% CIs overlapped the measured values. The model accurately recovered the ingestion rates of larval ABT on most mesozooplankton groups (Fig. 2b). The greatest model-data mismatch associated with larval ABT was for feeding on microzooplankton during C5 and feeding on poecilostomatoid copepods by preflexion larvae during both cycles. In all of these cases, none of the dietary items were found in the guts of the respective field-collected larvae (Shiroza *et al.*, this issue), while the model was constrained to take on positive values for all possible food-web fluxes. Model solutions were also strongly constrained by the comparatively low δ^15^N of larval ABT (Table I). The model struggled to determine solution vectors that matched the comparatively low δ^15^N of larval ABT with the fairly similar measured δ^15^N of upwelled nitrate, sinking detritus and bulk-suspended organic matter, thus leading to model misfits in the δ^15^N mass-balance equations.

### Food web dynamics

Food web dynamics broadly reflected those expected for an oligotrophic, recycling-dominant ecosystem. NH_4_^+^ was the dominant source of nitrogen to phytoplankton in the shallow euphotic zone (mean = 84%; 95% CI = 70–94% for C1 and 83%; 73–93% for C5). NO_3_^−^ uptake (13%, 4–25% for C1; 16%, 6–25% for C5) and N_2_ fixation (1.2%, 0.03–4.3% for C1; 1.2%, 0.03–4% for C5%) were comparatively less important. Nutrient utilization patterns were broadly similar in the vicinity of the deep chlorophyll maximum (>50 m depth), although they varied between the two cycles with nitrate becoming substantially more important in the deep euphotic zone during C1 (42%, 13–72% for C1) than during C5 (10%, 4–17% for C5). Total production was slightly higher in the shallow euphotic zone (2.4 mmol N m^−2^ d^−1^, 2.2–2.6 mmol N m^−2^ d^−1^ for C1; 3.5, 3.2–3.7 mmol N m^−2^ d^−1^ for C5) than in the deep euphotic zone (1.8 mmol N m^−2^ d^−1^, 1.7–1.9 mmol N m^−2^ d^−1^ for C1; 1.5, 1.4–1.6 mmol N m^−2^ d^−1^ for C5).

Most primary production was from picophytoplankton (54%, 40–68% for C1; 79%, 68–90% for C5) and flagellates (42%, 28–55% for C1; 21%, 14–28% for C5) in the shallow euphotic zone, as suggested by the field data. Diatoms were comparatively less important (4.9%, 3.2–6.8% for C1; 0.4%, 0.3–0.5% for C5), while *Trichodesmium* production was negligible. The relative proportions of each group were fairly similar at the deep chlorophyll maximum. Mixotrophic flagellates derived 18% (C1) and 24% (C5) of their nitrogen from phagotrophy in the shallow euphotic zone (and slightly more in the deep euphotic zone). They consumed more heterotrophic bacteria than cyanobacteria.

**Fig. 3:**
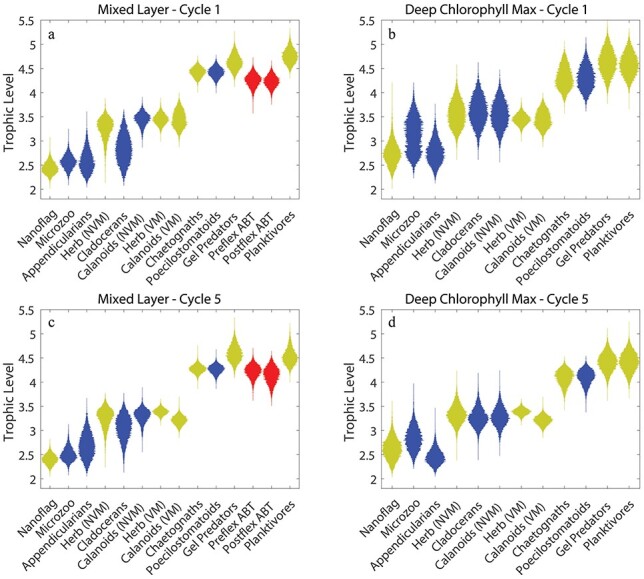
Violin plots of TL of zooplankton and fish in the mixed layer (**a**, **c**) and deep chl max (**b**, **d**) during C1 (a, b) and C5 (c, d). Blue plots are ABT prey. Red plots are ABT. Yellow plots are not ABT or their prey.

Phytoplankton mortality was dominated by protistan grazing. These zooplankton (including mixotrophic flagellates) consumed 64% (49–79%) of phytoplankton production during C1 and 54% (47–61%) during C5. Metazoan zooplankton consumed a lower portion of phytoplankton production (20%, 14–26% for C1; 23%, 16–30% for C5), although they consumed more of the production of diatoms than protists did. Suspension-feeding metazoans also relied heavily on protistan zooplankton as dietary sources. This was reflected in trophic positions that averaged greater than 3.0 for all metazoans except appendicularians (Fig. 3a–d). In the upper euphotic zone, predatory zooplankton (poecilostomatoid copepods, chaetognaths and gelatinous predators) had particularly high trophic positions of 4.4, 4.4 and 4.7, respectively, for C1 (Fig. 3a) and 4.3, 4.3 and 4.6 for C5 (Fig. 3c). Their mean trophic positions in the deep euphotic zone were similar (4.3, 4.3 and 4.6 for C1, Fig. 3b; and 4.1, 4.1 and 4.4 for C5, Fig. 3d, for poecilostomatoid copepods, chaetognaths and gelatinous predators, respectively).

**Fig. 4:**
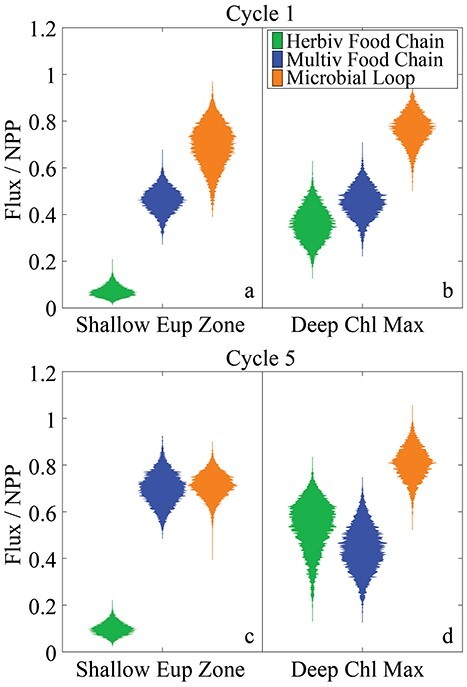
Violin plots of nitrogen flux through the herbivorous food chain (phytoplankton to metazooplankton), multivorous food chain (phytoplankton to metazooplankton via protistan grazers) and microbial loop (respiration from bacteria and protists supported by bacterial production) normalized to NPP for the shallow euphotic zone during C1 (**a**), deep euphotic zone during C1 (**b**), shallow euphotic zone during C5 **(c**) and deep euphotic zone during C5 (**d**).

Quantification of major food web pathways showed that the GoM euphotic zone is dominated by the microbial loop (Fig. 4). The microbial loop (defined as respiration by heterotrophic bacteria and the proportion of protistan respiration supported by bacterial production) processed 70% (51–90%) of NPP in the shallow euphotic zone (Fig. 4a) and 77% (61–91%) of the NPP in the deep euphotic zone (Fig. 4b) during C1, whereas during C5, it used 71%; 58–84% in the upper euphotic zone (Fig. 4c) and 81%; 65–96% in the lower euphotic zone (Fig. 4d). For comparison, the herbivorous and multivorous food chains were responsible for processing 7.2% and 46% of NPP, respectively, in the shallow euphotic zone (Fig. 4a), and 37 and 46%, respectively, in the deep euphotic zone, (Fig. 4b) during C1. During C5, the herbivorous and multivorous food chains were responsible for 9.8% and 70% of NPP in the shallow euphotic zone (Fig. 4c) and for 54% and 44% in the deep euphotic (Fig. 4d). The dominance of microbial loop pathways aligns with the importance of recycled NH_4_^+^ for phytoplankton production and conforms with an expectation of tight recycling in oligotrophic ecosystems with limited new nutrient supply. In the shallow euphotic zone, where recycling and the microbial loop were most important, DON production was substantial (2.0 and 2.9 mmol N m^−2^ d^−1^, for C1 and C5). Phytoplankton and protistan zooplankton had large roles in DON production (38 and 32%, respectively) during C1, with the remainder primarily coming from dissolution of detritus (9.8%) and mesozooplankton excretion (11%). During C5, phytoplankton exudation was responsible for 47% of DON production, while protists were responsible for 28% and metazoan zooplankton contributed 15% of DON production. Bacterial excretion was in turn responsible for 50% of NH_4_^+^ regeneration in the shallow euphotic zone during C1 and for 46% during C5, with protist excretion generating an additional 32 (C1) or 29% (C5) and mesozooplankton excretion producing 15% (C1) or 20% (C5) of the NH_4_^+^ used by phytoplankton.

### Larval Atlantic bluefin tuna in the GoM ecosystem

**Fig. 5:**
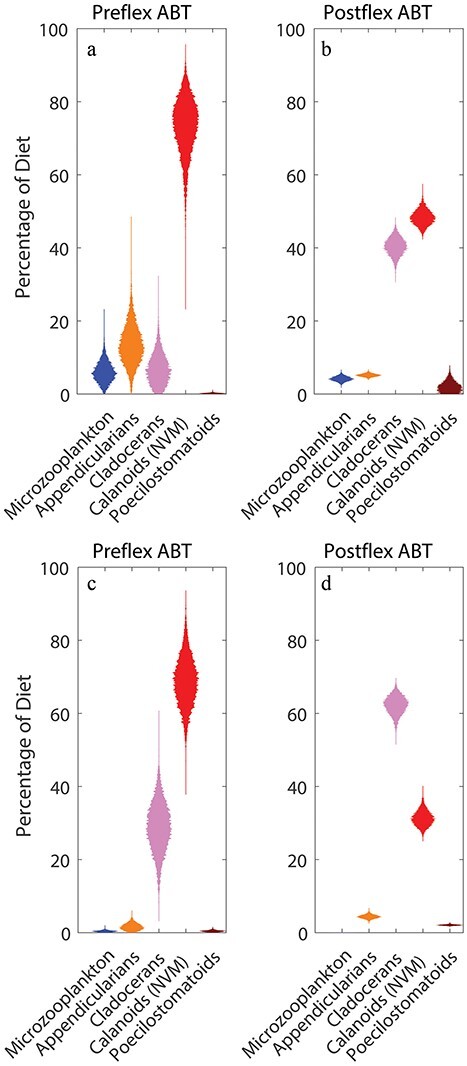
Violin plots of modeled larval ABT diets during C1 (**a**, **b**) and C5 (**c**, **d**).

As suggested by the gut content data, model results show that larval ABT feed predominantly on cladocerans and calanoid copepods, with a lesser role for microzooplankton, appendicularians and poecilostomatoid copepods in their diets (Fig. 5). Calanoid copepods comprised 76% of the diet of preflexion ABT (95% CI = 59–88%) during C1 and 69% (55–83%) during C5. Microzooplankton (6%; CI = 1–13% during C1; 0.4%, 0.03–0.9% during C5), appendicularians (14%; 4–26% during C1; 1.6%, 0.2–3.7% during C5) and cladocerans (4%; 0.3–9% during C1; 29%, 14–42% during C5) were smaller contributors to the diets of preflexion ABT, while poecilostomatoid copepods were negligible contributors to preflexion ABT diets (<0.7% during both cycles) (see Fig. 5a and c). Although calanoid copepods were also the dominant dietary source for postflexion ABT during C1 (62%; 59–66%), these larger larvae also relied substantially on cladocerans (23%; 19–26% during C1; 62, 57–67% during C5) (see Fig. 5b and d).

**Fig. 6:**
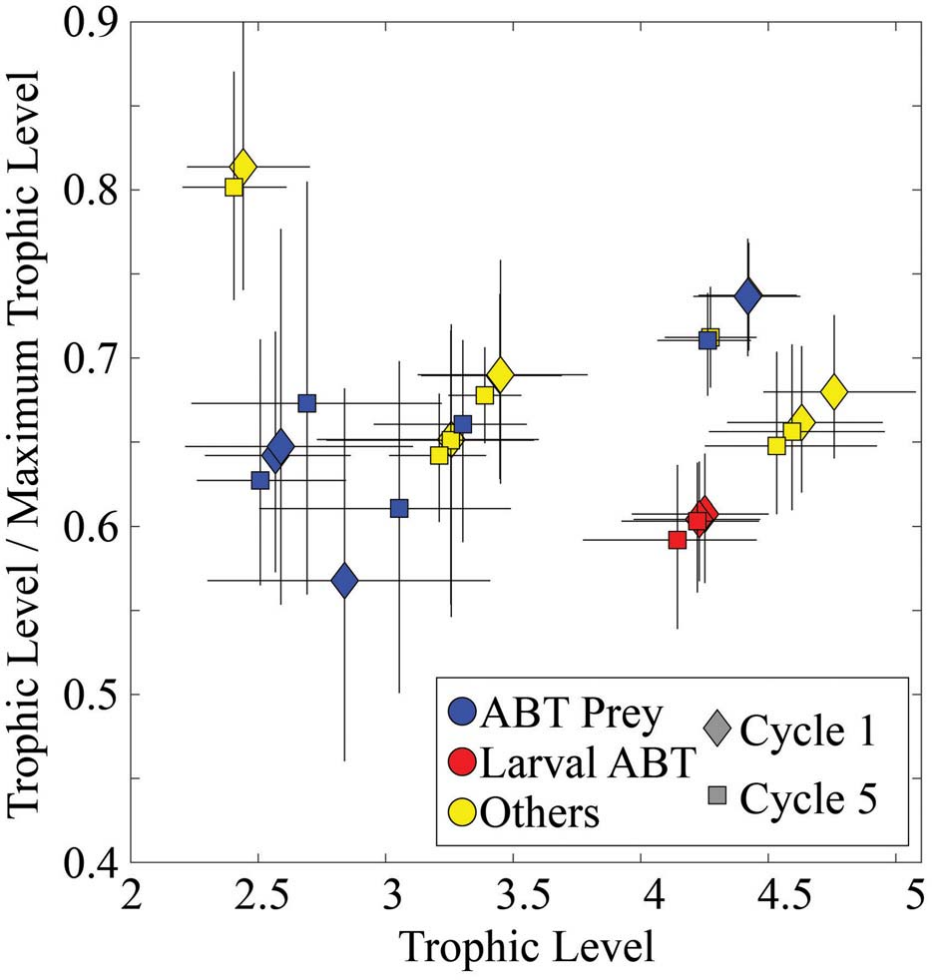
The ratio of the TL of different modeled zooplankton and fish to the TL they would have in the longest possible model food chain. The ‘other’ category includes all planktivorous fish and all zooplankton that are not larval ABT prey.

The prevalence of suspension-feeding zooplankton in the diets of both preflexion and postflexion ABT led to relatively low TLs for ABT larvae (Fig. 3a and c). Given the ecosystem structure used in the model (Fig. 1), larval ABT could potentially have a TL between 3 and 7. However, both preflexion and postflexion larvae had TLs on the low end of this range. Preflexion ABT had a TL of 4.2 (4.0–4.5) during C1 and 4.2 (3.9–4.5) during C5, while postflexion ABT had TL estimates of 4.2 (4.0–4.5) during C1 and 4.1 (3.8–4.5) during C5. Both developmental stages of ABT larvae thus had trophic positions averaging ~0.6 of their maximum possible TL (Fig. 6) and only one trophic position higher than their theoretically lowest possible TL within the food web. The trophic positions of larval ABT were thus notably low relative to those if feeding on the longest possible food chains that the model allowed. Based on this metric, their trophic positions were also notably lower than many of the zooplankton and other fish in the model.

**Fig. 7:**
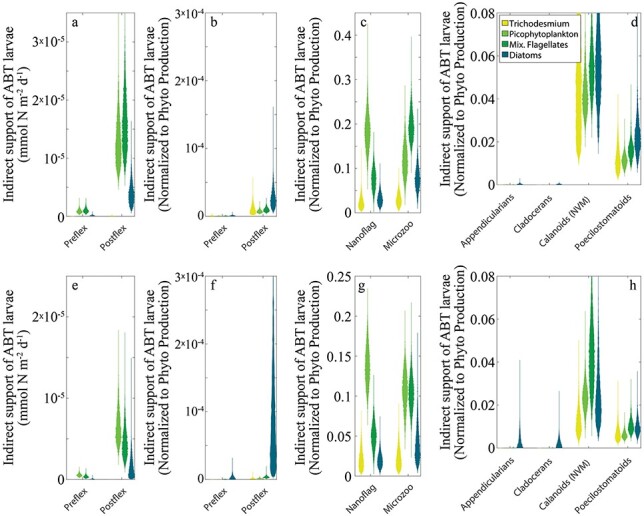
Indirect food web flows to larval tuna (**a**, **b**, **e**, **f**), protists (**c**, **g**) and mesozooplankton (**d**, **h**). Panels (a) and (e) show the amount of organic matter derived from each phytoplankton taxon that was respired by larval tuna. Other panels show the proportion of the production of each phytoplankton taxon that was respired by either larval tuna (b, f), protists (c, g) or mesozooplankton (d, h). Only ABT prey are shown in (d) and (h). Panels (a–d) are for C1; (e–h) are for C5.

The food chains supporting larval ABT were diverse and relied on significant production of picophytoplankton, flagellates and diatoms (while the production of *Trichodesmium* was insignificant for ABT food chains). Preflexion ABT excreted 1.0 (0.4–2.0) nmol N m^−2^ d^−1^ derived from the production of flagellates, 0.9 (0.4–1.8) nmol N m^−2^ d^−1^ from picophytoplankton and 0.16 (0.05–0.37) nmol N m^−2^ d^−1^ from diatoms during C1 (Fig. 7a). During C5, preflexion ABT excreted 0.37 (0.14–0.79), 0.57 (0.28–0.99) and 0.06 (0.005–0.21) nmol N m^−2^ d^−1^ from flagellates, picophytoplankton and diatoms, respectively (Fig. 7e). Postflexion larvae excreted 17 (8–29) nmol N m^−2^ d^−1^ from flagellates, 13 (6.7–23) nmol N m^−2^ d^−1^ from picophytoplankton and 4.1 (1.2–9.2) nmol N m^−2^ d^−1^ from diatoms during C1 (Fig. 7a) and 4.7 (1.8–9.7), 6.7 (3.4–12) and 1.8 (0.14–5.3) nmol N m^−2^ d^−1^ during C5 (Fig. 7e). These values were influenced in large part by the different production rates of each phytoplankton taxa (flagellate, picophytoplankton and diatom NPP in the shallow euphotic zone were 1.8, 1.7 and 0.24 mmol N m^−2^ d^−1^ during C1 and 1.5, 4.2 and 0.015 mmol N m^−2^ d^−1^ during C5). When normalized to phytoplankton NPP, it becomes clear that larval ABT rely disproportionately on the production of large phytoplankton (Fig. 7b and f), even though large phytoplankton production is low in absolute terms. Preflexion ABT respired 1.1 × 10^−4^% (C1) and 4.0 × 10^−4^% (C5) of diatom NPP and 6.3 × 10^−5^% (C1) and 2.9 × 10^−5^% (C5) of flagellate NPP when compared to only 5.2 × 10^−5^ %(C1) and 1.5 × 10^−5^% (C5) of picophytoplankton NPP. Postflexion larvae respired 2.9 × 10^−3^% (C1) and 1.1 × 10^−2^% (C5) of diatom NPP, 1.0 × 10^−3^% (C1) and 3.7 × 10^−4^% (C5) of flagellate NPP and 7.5 × 10^−4^% (C1) and 1.8 × 10^−4^% (C5) of picophytoplankton NPP. The proportion of *Trichodesmium* NPP respired by larvae was poorly constrained by the model, although *Trichodesmium* production was consistently low in all model solution vectors. The disproportionately large role of diatoms in larval ABT diets was reflected in the roles of diatoms in supporting their mesozooplankton prey (Fig. 7d and h). Three of the four mesozooplankton prey taxa respired a higher proportion of diatom NPP than any other phytoplankton, while calanoids relied slightly more on flagellates than on diatoms during C5 (although they also preferentially relied on diatoms during C1). These results for mesozooplankton were in stark contrast to similar proportional roles for phytoplankton in protistdiets (Fig. 7c and g). Heterotrophic nanoflagellates relied disproportionately on picophytoplankton, respiring 19% of picophytoplankton NPP during C1 (14% during C5), while microzooplankton relied disproportionately on the NPP of flagellates (respiring 20% of flagellate NPP during C1 and 11% during C5).

### Nitrogen cycle and support of the upper euphotic zone ecosystem

In nitrogen-limited ecosystems, such as the open-ocean GoM, the supply of new nitrogen can control overall ecosystem productivity. Our results suggest that in the upper euphotic zone where ABT larvae feed, the ecosystem is not substantially supported by locally upwelled nitrate (which supplied 4.4 × 10^−5^ (2.1 × 10^−5^–8.4 × 10^−5^) mmol N m^−2^ d^−1^ to the upper euphotic zone during C1 and 4.3 × 10^−5^ (2.3 × 10^−5^–8.4 × 10^−5^) mmol N m^−2^ d^−1^ during C5) or by nitrogen fixation [which supplied 0.092 (4.3 × 10^−3^–0.32) mmol N m^−2^ d^−1^ to the upper euphotic zone during C1 and 0.06 (0.002–0.2%) mmol N m^−2^ d^−1^ during C5]. Rather, nitrogen entered the ecosystem primarily through the lateral advection of organic matter (PON lateral advection = 0.18, 0.007–0.51 mmol N m^−2^ d^−1^ during C1; 0.94, 0.30–1.6 mmol N m^−2^ d^−1^ during C5; DON lateral advection = 0.19, 0.007–0.45 mmol N m^−2^ d^−1^ during C1; 0.10, 0.002–0.37 mmol N m^−2^ d^−1^ during C5) from more productive regions (likely from shelf-break regions in the northern GoM, Gerard *et al.*, this issue). Indeed, ABT derived only 0.2% (0.004–0.7%) of their nitrogen from upwelled nitrate during C1 and 0.1% (0.003–0.46%) during C5 and 2.2% (0.2–7.6%) and 0.95% (0.08–3.3%) of their nitrogen from nitrogen fixation during C1 and C5, respectively. They derived 98% (92–>99%) and 99% (97–>99%) from lateral advection.

This laterally advected organic matter entered the planktonic food web through multiple pathways. DON was utilized by bacteria, which had a gross growth efficiency of 27% (20–30%) during C1 and 28% (24–30%) during C5 and hence converted 73% of the DON they utilized to NH_4_^+^ during C1 and 72% during C5. The suspended particles (which presumably comprised laterally advected PON) were consumed primarily by protistan grazers or were converted into DON (likely through the activity of particle-attached microbes that were not explicitly included in our model). This highlights the importance of the microbial food web in mediating and enhancing phytoplankton in oligotrophic regions. Indeed, even nitrate (which is often considered to be a ‘new’ nutrient in the euphotic zone) was primarily produced *in situ* by microbial activity (i.e. nitrification conducted by implicitly modeled ammonium-oxidizing bacteria). Modeled nitrification rates in the upper euphotic zone were 0.45 (0.15–0.83) mmol N m^−2^ d^−1^ during C1 and 0.08 (0.3–1.3) mmol N m^−2^ d^−1^ during C5. This equates to nitrification rates of 8.9 and 16 nmol N L^−1^ d^−1^ for C1 and C5, respectively. Notably, despite these low absolute nitrification rates, nitrate in the surface ocean was able to be regenerated every 2–3 days because nitrate concentrations were consistently low in the upper euphotic zone. Considering that ammonium concentrations were ~90 nmol L^−1^ during C1 and ~50 nmol L^−1^ during C5, this suggests a specific ammonium-oxidation rate of 0.1 d^−1^ during C1 and 0.32 d^−1^ during C5. These results highlight the complexity of microbial and zooplankton linkages that support larval ABT in their oligotrophic nursery regions and suggest that the circulation of the GoM plays an important role in sustaining suitable conditions for larval growth.

## DISCUSSION

The open-ocean GoM is an incredibly oligotrophic ecosystem with low productivity and a deep nitracline (Biggs, 1992; Gomez *et al.*, 2018; Knapp *et al.*, this issue; Yingling *et al.*, this issue). Nevertheless, it is an important spawning ground for many migratory fish species, including multiple species of tuna, dolphinfish, sailfish and marlin (Rooker *et al.*, 2012; Kitchens and Rooker, 2014; Cornic *et al.*, 2018; Laiz-Carrión *et al.*, 2019). It is also a region in which substantially depressed vertical mixing limits the phytoplankton productivity during ABT spawning periods (Gomez *et al.*, 2018). If nutrient supply is indeed crucial for supporting these oligotrophic systems, predicted future warming and increased stratification could have deleterious impacts on taxa living in the mixed layer (Muhling *et al.*, 2011; Liu *et al.*, 2015; Muhling *et al.*, 2015). Understanding how pelagic ecosystems and the larval fish they support will respond to climate change requires knowledge of the food web pathways that convert phytoplankton production into the preferred prey of different species (Landry *et al.*, 2019).

We can hypothesize two potential ways in which an organism’s diet could make it well adapted to life in an oligotrophic region. First, it could feed preferentially on taxa that have either direct or indirect linkages to some of the most abundant primary producers in the ecosystem (e.g. cyanobacteria). For instance, a reliance on appendicularians would give larval fish access to a suspension feeder that can consume picophytoplankton directly (Gorsky and Fenaux, 1998; Llopiz *et al.*, 2010). Conversely, preference for calanoid copepods and podonid cladocerans would make a larval fish more dependent on the production of diatoms and other large phytoplankton. A second, but not mutually exclusive, hypothesis is that larval fish are more likely to thrive in oligotrophic ecosystems if they feed at a low trophic position, thus maximizing trophic transfer efficiency from phytoplankton to larvae regardless of the source of production.

Our results provide no evidence for the former hypothesis. Although diatom production only contributed to ~10% of ABT larval diets, it was a disproportionately high fraction of the ABT diet relative to the proportional role of diatoms to total NPP in the upper euphotic zone (~5% during C1, <1% during C5). Indeed, relative to a phytoplankton taxon’s productivity, the proportional contribution of each phytoplankton taxon to food-web pathways that support pre- and postflexion ABT larvae increased with increasing phytoplankter size from picophytoplankton to flagellates to diatoms (Fig. 7). The disproportionately large role of diatom-driven pathways was largely the result of the important role that podonid cladocerans played in ABT diets. Although they were only abundant in the water column during C5, our experiment conducted closest to the shelf break, podonids were consistently over-represented in ABT guts (Shiroza *et al.*, this issue). Cladocerans are more frequently found in the coastal regions of the GoM, suggesting that they prey preferentially on large phytoplankton, as suggested by the LIEM and consistent with feeding studies (Kim *et al.*, 1989; Katechakis and Stibor, 2004). Non-vertically migrating calanoid copepods, which formed another important component of ABT diets (because they were the most abundant mesozooplankton prey available to ABT), had a more diverse diet of diatoms, mixotrophic flagellates and heterotrophic protists. By contrast, while efficient pathways from cyanobacteria to ABT larvae can occur through appendicularians and microzooplankton, these taxa were not abundant in ABT guts. Appendicularians were rare in the water column, while microzooplankton were abundant but were seldom selected by ABT. The majority of cyanobacteria were consumed by heterotrophic nanoflagellates. These heterotrophic nanoflagellates had moderate gross growth efficiency in the model (30–36%) and were preyed upon by other protists (microzooplankton) and suspension-feeding metazoans. Cyanobacteria and heterotrophic nanoflagellates thus contributed disproportionately to the recycling pathways of the microbial loop, forming a largely distinct food web from the multivorous and herbivorous pathways, which mostly began with mixotrophic flagellates and diatoms and supported the production of larval ABT and other planktivorous fish. Despite distinct differences in prey selectivity with ontogeny (large cladocerans were much more important prey for postflexion larvae, Shiroza *et al.*, this issue), our results show similar dependence on large phytoplankton for both larval stages.

Our results offer more support for the hypothesis that ABT larvae feed at a relatively low TL, maximizing the proportion of NPP available to them, and help explain how they survive in their oligotrophic spawning grounds (Fig. 6). The trophic position of ABT larvae (~4) is much closer to the minimum TL that our model allows (3: phytoplankton→prey→larvae) than to the maximum allowed TL (7: phytoplankton→bacteria→nanoflagellates→microzooplankton→suspension-feeders →carnivorous zooplankton→larvae). The low trophic position of ABT larvae is particularly striking, considering the relatively weak herbivorous food chain. Generally, planktivorous fish are more likely to be at a low TL in an ecosystem classically dominated by large phytoplankton and herbivorous mesozooplankton. However, the herbivorous food chain was responsible for only 7.2% (C1) or 9.8% (C5) of NPP processing in the shallow euphotic zone where the ABT larvae feed; the multivorous food chain processed 46% (C1) or 70% (C5) of NPP, while the microbial loop processed 70–71% of NPP on both cycles (Fig. 4, and note that the total exceeds 100% because NPP does not include phytoplankton DON production). The low trophic positions of ABT larvae were primarily due to two factors: (i) although total protistan secondary production was higher than total mesozooplankton secondary production, a comparatively small proportion of this secondary production made its way to larval tuna; most was dissipated as respiration in the microbial loop. Food chains supporting larval ABT were largely distinct from those involving the smallest class of heterotrophic protists. (ii) Both size classes of ABT larvae fed preferentially on podonid cladocerans, which fed lower in the food chain than other suspension-feeding taxa. Shiroza *et al.* (this issue) found selection for cladocerans to be an active process, further supporting the notion that ABT larvae are highly specialized at maximizing trophic efficiency within their oligotrophic nurseries.

While the trophic position of ~4 is low for a species known to preferentially feed on carnivorous copepods (poecilostomatoids) in a cyanobacteria- and microbial loop-driven ecosystem, we note that this is not actually a low TL relative to some other mass-balance constrained marine food web models. Many models based on ECOPATH software include only one (or zero) protistan trophic step and a single mesozooplankton group (Arreguin-Sanchez *et al.*, 2004; Walters *et al.*, 2008; Geers *et al.*, 2016). These models constrain zooplankton to TLs 2 or 3; hence, the maximum allowed trophic position for planktivores is only 3 or 4. The additional complexity of our modeled ecosystem is a far more realistic depiction of natural food web complexity (Fig. 1). Even so, our model allows only a maximum of two trophic steps within the protistan zooplankton (heterotrophic nanoflagellates and microzooplankton), which is an arbitrary limit, given the fluidity of protistan trophic interactions (Boenigk and Arndt, 2002; Pomeroy *et al.*, 2007; Calbet, 2008; Caron *et al.*, 2012; Sherr and Sherr, 2016). Some protists (e.g. pallium-feeding dinoflagellates) routinely feed at a 1:1 predator:prey size ratio, while others (e.g. ciliates) feed closer to a 10:1 predator:prey size ratio (Kiørboe, 2008; Fuchs and Franks, 2010). Some protists may consequently function at a higher trophic position than allowed by our model.

The BLOOFINZ–GoM study offers new insights to the physical dynamics of the GoM that support larval ABT. Kelly *et al*. (in review) analyzed vertical profiles of nitrate and buoyancy frequency from our cruises and concluded that exceedingly low amounts of nitrate were upwelled into the shallow euphotic zone where ABT spawn and their larvae grow. Instead, results from remote-sensing products and a 3D biogeochemical model provide compelling evidence that most nitrogen for export in the ABT habitat arrives via horizontal advection of organic matter. While our model constrains these inputs to be non-living organic matter (PON and DON), we note that a substantial proportion of this organic matter might be living plankton advected from more productive regions including the shelf-break region of the northern GoM and the Campeche Banks region north of the Yucatan Peninsula. Indeed, Gerard *et al*. (this issue) backtracked physical flows for the source of waters sampled in C1 and C5 to their origins 2–4 weeks previously along the shelf-slope margin in the northeastern GoM. Stukel *et al*. (this issue) found that ~20% of particulate organic carbon in the upper euphotic zone was contained in living organisms. Landry and Swalethorp (this issue) further determined that (particularly during C5) predatory zooplankton likely relied on prey advected into our study region from more productive areas. Shropshire *et al*. (this issue) showed that ABT survival was also dependent on advection of prey from coastal areas and concluded that the most beneficial region for ABT spawning was near the shelf-break where prey are abundant for first-feeding larvae, but where offshore currents can transport larvae that survive the critical period to more oligotrophic regions before predation becomes a substantial source of mortality.

Our results show the importance of extensive recycling pathways for supporting phytoplankton production in this habitat. Despite the very low rates of vertical nitrate input and nitrogen fixation to the upper euphotic zone, sinking carbon flux from the upper euphotic zone was substantial (Stukel *et al.*, this issue). This export, and indeed nearly all production in the upper euphotic zone, was supported by nutrients regenerated from PON through the activity of heterotrophic bacteria and protistan zooplankton. NH_4_^+^ was responsible for ~85% of the production of phytoplankton in the upper euphotic zone, as is commonly the case in the mixed layer of oligotrophic, open-ocean regions (McCarthy *et al.*, 1996; Lipschultz, 2001). However, in contrast to simple interpretations of nitrogen utilization, even NO_3_^−^ was primarily generated autochthonously in the shallow euphotic zone and did not represent a ‘new’ form of nitrogen. The utility of nitrate as a tracer of ‘new’ production (Eppley and Peterson, 1979) has been extensively debated in the light of evidence of nitrification in shallow waters (Yool *et al.*, 2007). The emerging consensus suggests that ammonium-oxidizing bacteria are not intrinsically light-inhibited (although some taxa may be), but rather they are often outcompeted in the euphotic zone by *Prochlorococcus* and other low-nutrient specialist phytoplankton (Smith *et al.*, 2014; Wan *et al.*, 2018; Xu *et al.*, 2019). Our results do not contradict this view. Indeed, the LIEM suggests that phytoplankton utilize NH_4_^+^ more rapidly than ammonium-oxidizing bacteria. However, the low NO_3_^−^ concentrations throughout the euphotic zone (Knapp *et al.*, this issue) and exceedingly low NO_3_^−^ flux (Kelly *et al.*, in review) allow nitrification to dominate NO_3_^−^ supply despite low absolute nitrification rates. Indeed, our estimate of the specific rate of ammonium oxidation necessary to support phytoplankton NO_3_^−^ utilization (0.1–0.3 d^−1^) is near the median value for surface ocean ammonium oxidation in the synthesis of Yool *et al*. (2007). Notably, Clark *et al*. (2008) measured ammonium and nitrite oxidation rates in the oligotrophic regions of the Atlantic Ocean which were slightly lower than our LIEM-predicted values, and Bronk *et al*. (2014) measured substantially higher nitrification rates in the offshore regions of the West Florida Shelf. Nitrification rate measurements from other regions have been highly variable, and there is not, as yet, a consensus on the relative importance of shallow nitrification to NO_3_^−^ supply in oligotrophic regions (Newell *et al.*, 2013; Clark *et al.*, 2016; Shiozaki *et al.*, 2016).

The importance of laterally advected organic matter for supporting oligotrophic communities in the GoM offers important insight into the physical characteristics that make the GoM an ideal spawning habitat for ABT. While previous studies have focused on the role of vertical mixing and upwelling, our results show that mixed layer productivity may be more directly tied to horizontal fluxes driven by the high mesoscale energy of the GoM. In the oligotrophic GoM, the Loop Current and the eddies that it sheds are prominent features enhancing circulation (Forristall *et al.*, 1992; Oey *et al.*, 2005; Schmitz *et al.*, 2005). These features have the potential to fundamentally restructure open-ocean ecosystems, with warm-core eddies (including Loop Current Eddies) depressing the nutricline and primary production, while cold-core eddies increase open-ocean upwelling and productivity (Biggs and Müller-Karger, 1994). These altered nutrient supply and phytoplankton regimes lead to substantially higher zooplankton biomass in cold-core eddies (Wells *et al.*, 2017). However, the relative importance of each eddy type, as well as the distinct gradient regions that form on their edges, on larval ABT remains a topic of active debate (Muhling *et al.*, 2010; Domingues *et al.*, 2016). Our results suggest that both eddy types can be important nitrogen sources to the upper euphotic zone since the high horizontal velocities along the eddy can transport living and non-living organic matter from high biomass regions to oligotrophic areas, especially when eddies impinge on coastal regions. Shropshire *et al*. (2020) also found substantial transport into our study region mediated by entrainment of plankton-rich waters from the Campeche Bank into the edges of the Loop Current. Notably, the larvae distribution in the major recognized ABT eastern stock spawning area, around the Balearic Islands in the western Mediterranean basin, is influenced by frontal zones resulting from the convergence of recent and resident Atlantic surface waters (Alemany *et al.*, 2010; Muhling *et al.*, 2017; Reglero *et al.*, 2017). Such mesoscale features have been hypothesized to act as retention larval feeding areas, enhancing particle food concentrations and increasing the probability of survival of larvae that rely substantially on copepodites and cladoceran prey during preflexion stage in this oligotrophic environment (Catalán *et al.*, 2011; Uriarte *et al.*, 2019). Horizontal flows associated with these features may also connect the nearby coastal region to oligotrophic nursery areas, a possibility that should be explored in future studies.

The potential importance of cross-shore fluxes to survival of first-feeding ABT suggests that determining the responses of pelagic food webs and ABT larvae to climate change will require characterizing changes in GoM circulation in response to future forcing along with the expected food web processes that regenerate nutrients and promote growth of larval ABT prey (Muhling *et al.*, 2011; Liu *et al.*, 2015). Our study offers insight into the processes allowing larval ABT to survive in a food-scare environment. However, substantial additional research is needed to quantify the impacts of spatial and interannual variability, as well as secular change, on these ecosystems and threatened species.

## CONCLUSION

ABT larvae develop in oligotrophic ecosystems, dominated by cyanobacteria and other small phytoplankton. The major trophic pathway through the microbial loop is highly inefficient, with most production lost to remineralized nutrients by bacteria and multi-step protistan grazing chains. Both pre- and postflexion larval ABT feed preferentially on less dominant pathways associated with herbivorous and multivorous food chains, without pronounced ontogenetic differences in food-web roles between pre- and postflexion stages, despite distinct changes in diet. Consequently, ABT larvae depend on the production of diatoms and mixotrophic flagellates that support herbivorous zooplankton, particularly calanoid copepods and cladocerans. Preferential utilization of these more direct trophic pathways allows the larvae to feed at relatively low TLs despite the fact that the taxa responsible for the majority of secondary production in the food web (bacteria and heterotrophic nanoflagellates) are not accessible to them as prey. Further research is needed to understand how these ecological interactions might be altered under different disturbance regimes.

## DATA AVAILABILITY

Data utilized in this manuscript are available on NCCOS and BCO-DMO (https://www.bco-dmo.org/project/819488). MCMC+^15^N model code is available on Github: https://github.com/stukel-lab. The specific code used to run the BLOOFINZ-GoM inverse model, with set-up files and instructions for running it can be accessed at: https://github.com/mstukel/N15-LIM-BLOOFINZ-GoM

## Supplementary Material

Stukel_et_al_GoM_BFT_Inverse-Draft_6_3-supplement_fbab023
