# Supplementary material for: Plankton food webs in the oligotrophic Gulf of Mexico spawning grounds of Atlantic bluefin tuna
Source: J Plankton Res. 2021 Apr 22;44(5):763–81. doi: 10.1093/plankt/fbab023 (PMC9424712; doi:10.1093/plankt/fbab023)
Supplement: Stukel_et_al_GoM_BFT_Inverse-Draft_6_3-supplement_fbab023 [file stukel_et_al_gom_bft_inverse-draft_6_3-supplement_fbab023.docx]

**Supplementary Material for:**

**Plankton food webs in the oligotrophic Gulf of Mexico spawning grounds of Atlantic Bluefin tuna**

**Michael R. Stukel^1,2,*^, Trika Gerard^3^, Thomas Kelly^1^, Angela N. Knapp^1^, Raúl Laiz-Carrión^4^, John T. Lamkin^3^, Michael R. Landry^5^, Estrella Malca^6^, Karen E. Selph^7^, Akihiro Shiroza^6^, Taylor A. Shropshire^1,2^, Rasmus Swalethorp^5^**

^1^ EARTH, OCEAN AND ATMOSPHERIC SCIENCE, FLORIDA STATE UNIVERSITY, TALLAHASSEE, FL 32306, USA

^2^ CENTER FOR OCEAN-ATMOSPHERIC PREDICTION STUDIES, FLORIDA STATE UNIVERSITY, TALLAHASSEE, FL 32306, USA

^3^SOUTHEAST FISHERIES SCIENCE CENTER, NATIONAL MARINE FISHERIES SERVICE, NATIONAL OCEANIC AND ATMOSPHERIC ADMINISTRATION (NOAA), MIAMI, FL 33149, USA

^4^CENTRO OCEANOGRÁFICO DE MALAGA, INSTITUTO ESPAÑOL DEL OCEANOGRAFÍA, FUENGIROLA, SPAIN

^5^ SCRIPPS INSTITUTION OF OCEANOGRAPHY, 9500 GILMAN DR., LA JOLLA, CA 92093-0227, USA

^6^ COOPERATIVE INSTITUTE FOR MARINE AND ATMOSPHERIC STUDIES, UNIVERSITY OF MIAMI, MIAMI, FL 33149, USA

^7^ DEPARTMENT OF OCEANOGRAPHY, UNIVERSITY OF HAWAII AT MANOA, HONOLULU, HI 96822, USA

*CORRESPONDING AUTHOR: [mstukel@fsu.edu](mailto:mstukel@fsu.edu)

**Supplementary Text – Online Appendix 1**

**Inverse model implementation**

To constrain the flux of nitrogen through unmeasured ecosystem pathways, we used linear inverse ecosystem modeling (LIEM) techniques ([Vézina and Platt, 1988](#_ENREF_10); [Van Oevelen *et al.*, 2010](#_ENREF_9)). LIEM allows investigators to specify mass balance constraints that must be exactly fit by food web solutions ($\boldsymbol{A}\vec{x}=\vec{b}$), approximate equations that quantify measured rates with associated measurement uncertainty ($\boldsymbol{E}\vec{x}\approx\vec{f}$), and inequality constraints ($\boldsymbol{G}\vec{x}\geq\vec{h}$) that represent *a priori* acceptable ranges for different ecosystem properties. In these equations, $\vec{x}$ represents the vector of food web flows that must be solved. For instance, in our model the first food web flux represents upwelling of nitrate from the deep euphotic zone to the shallow euphotic zone. Other food web fluxes include such things as nitrogen fixation by *Trichodesmium* in the upper euphotic zone, grazing of appendicularians on heterotrophic bacteria in the lower euphotic zone, ammonium excretion by preflexion ABT, and sinking flux of large detritus leaving the lower euphotic zone. Our model includes a total of 302 food web flows to be solved for (i.e., $\vec{x}$ encodes 302 food web fluxes, see Fig. 1 and Supp. Table 1).

In the equation $\boldsymbol{A}\vec{x}=\vec{b}$, the matrix $\boldsymbol{A}$ encodes mass balance constraints for all 44 model compartments (e.g., nitrate in the upper euphotic zone; poecilostomatoid copepods in the lower euphotic zone) and $\vec{b}$ is a vector of zeroes, because we assume that the ecosystem is at steady state. These mass balance constraints will be exactly fit by all solution vectors, as explained below.

The approximate equation $\boldsymbol{E}\vec{x}\approx\vec{f}$ encodes two types of information. The first is direct in situ field measurements that are connected to distinct model flows. For instance, protistan grazing on cyanobacteria measured in the upper euphotic zone by the dilution approach ([Landry *et al.*, this issue](#_ENREF_2)) is equal to the sum of heterotrophic nanoflagellates grazing on cyanobacteria + microzooplankton grazing on cyanobacteria + mixotrophic flagellates grazing on cyanobacteria in this layer. The model included 38 such direct measurement constraints. Because these rates are measured with uncertainty, the model is not forced to match them exactly.

The other type of data encoded within $\boldsymbol{E}\vec{x}\approx\vec{f}$ are mass balance constraints for flows of ^15^N for each compartment (44 additional approximate equalities). Because fluxes of ^15^N through the ecosystem are impacted by isotopic fractionation with uncertain fractionation coefficients, we incorporate these mass balance constraints using approximate equality constraints, rather than exact equality constraints ([Stukel *et al.*, 2018a](#_ENREF_5); [Stukel *et al.*, 2018b](#_ENREF_6)).

The inequality $\boldsymbol{G}\vec{x}\geq\vec{h}$ encodes known constraints on organisms and/or the ecosystem as a whole (e.g., gross growth efficiency of zooplankton is between 10% and 40%, all food web fluxes must be positive, respiration must be greater than a temperature-dependent function of biomass). All model solutions must fit within these greater than/less than constraints. We included 533 such inequality constraints. Note that all of the exact equalities, inequalities, and approximate equalities can be found (along with the code needed to run the model) in a GitHub repository (<https://github.com/mstukel/N15-LIM-BLOOFINZ-GoM>). The inequalities, equalities, and approximate qualities specifically are found in the excel file ‘N15InverseModelRW.GoM.xlsx’ in the repository.

Despite the large number of constraints included in this modeling study, with 302 total unknown food web flows ($\vec{x}$), the system remains under-constrained. To objectively determine a representative solution (and confidence limits), we used the Markov Chain Monte Carlo (MCMC) with ^15^N approach ([Stukel *et al.*, 2018a](#_ENREF_5); [Stukel *et al.*, 2018b](#_ENREF_6)). The MCMC approach initially uses the exact mass balance constraints ($\boldsymbol{A}\vec{x}=\vec{b}$) to remove degrees of freedom from the solution and then creates bounds on the solution as formed by the hyperplanes prescribed by the inequality constraints ($\boldsymbol{G}\vec{x}\geq\vec{h}$) ([Kones *et al.*, 2009](#_ENREF_1); [Soetaert *et al.*, 2009](#_ENREF_4); [Van Den Meersche *et al.*, 2009](#_ENREF_8)). Then, starting with an initial guess of the solution that satisfies the equality and inequality constraints, the MCMC approach conducts a random walk through the solution space bounded by $\boldsymbol{A}\vec{x}=\vec{b}$ and $\boldsymbol{G}\vec{x}\geq\vec{h}$. New solutions are accepted based on the relative misfits of the new and previous solution with respect to the approximate equality measurements ($\boldsymbol{E}\vec{x}\approx\vec{f}$) and the uncertainty associated with the measurements codified in this approximate equation ([Van Den Meersche *et al.*, 2009](#_ENREF_8); [Stukel *et al.*, 2018a](#_ENREF_5)). This generates a set of solutions satisfying the equality and inequality constraints. The probability of inclusion for a specific solution is related to how well it satisfies the combined field measurement and ^15^N mass balance constraints. The arithmetic mean solution of the MCMC approach has been shown to more accurately recover withheld measurement constraints than the previously used L_2_ minimum norm approach ([Stukel *et al.*, 2012](#_ENREF_7); [Saint-Béat *et al.*, 2013](#_ENREF_3)). The MCMC+^15^N approach used herein builds on this previous work, but allows for the incorporation of non-linear constraints associated with unknown δ^15^N values for some organisms or non-living nitrogen pools in the ecosystem to further constrain the system. This approach uses a second varying solution vector ($\vec{\delta}$) quantifying the ^15^N isotope fraction for each unknown nitrogen pool. A new solution set for $\vec{\delta}$ is determined at the same time as the new solution set for $\vec{x}$. $\vec{\delta}$ modifies the 44 approximate equality constraints that are associated with ^15^N mass balance. For additional details, see Stukel *et al*. ([2018a](#_ENREF_5)).

For the model implementation used in this study, we included “weights” for different model flows that were related to the expected magnitude of each flow (i.e., we multiplied $\vec{x}$ by a vector of weights and divided the rows of $\boldsymbol{A}$, $\boldsymbol{G}$, and $\boldsymbol{E}$ by the same vector of weights). This was done to approximately normalize the magnitude of all flows in $\vec{x}$ to maximize computational efficiency and was necessary because some flows (e.g., ammonium uptake by cyanobacteria) were many orders of magnitude larger than other flows (e.g., preflexion ABT feeding on poecilostomatoid copepods). For each cycle, we computed approximately 100 million solution vectors during the MCMC+^15^N procedure. We then thinned the solution set by only retaining every 10,000^th^ solution vector to avoid autocorrelation, and removed the first 20% of the solution vectors (i.e., the first 20 million solution vectors) as a burn-in period. Jump lengths (e.g., the distance traveled from one solution vector to the next proposed solution vector) were tuned to ensure approximately 30% acceptance rates of new solutions.

**References**

Kones, J. K., Soetaert, K., Van Oevelen, D. and Owino, J. O. (2009) Are network indices robust indicators of food web functioning? A Monte Carlo approach. *Ecological Modelling,* 220, 370-382.

Landry, M. R., Selph, K. E., Stukel, M. R., Swalethorp, R., Kelly, T. B., Beatty, J. and Quackenbush, C. R. (this issue) Microbial food web dynamics in the oceanic Gulf of Mexico. *J. Plankton Res.*

Saint-Béat, B., Vézina, A. F., Asmus, R., Asmus, H. and Niquil, N. (2013) The mean function provides robustness to linear inverse modelling flow estimation in food webs: A comparison of functions derived from statistics and ecological theories. *Ecological Modelling,* 258, 53-64.

Soetaert, K., Van Den Meersche, K. and Van Oevelen, D. (2009) limSolve: Solving linear inverse models*R package version.* Vol. 1.5.4.

Stukel, M. R., Décima, M. and Kelly, T. B. (2018a) A new approach for incorporating 15N isotopic data into linear inverse ecosystem models with Markov Chain Monte Carlo sampling. *PloS one,* 13, e0199123.

Stukel, M. R., Décima, M., Landry, M. R. and Selph, K. E. (2018b) Nitrogen and isotope flows through the Costa Rica Dome upwelling ecosystem: The crucial mesozooplankton role in export flux. *Global Biogeochem. Cycles,* 32, 1815-1832.

Stukel, M. R., Landry, M. R., Ohman, M. D., Goericke, R., Samo, T. and Benitez-Nelson, C. R. (2012) Do inverse ecosystem models accurately reconstruct plankton trophic flows? Comparing two solution methods using field data from the California Current. *J. Mar. Sys.,* 91, 20-33.

Van Den Meersche, K., Soetaert, K. and Van Oevelen, D. (2009) xSample(): An R function for sampling linear inverse problems. *Journal of Statistal Software, Code Snippets,* 30, 1-15.

Van Oevelen, D., Van Den Meersche, K., Meysman, F. J. R., Soetaert, K., Middelburg, J. J. and Vezina, A. F. (2010) Quantifying food web flows using linear inverse models. *Ecosystems,* 13, 32-45.

Vézina, A. F. and Platt, T. (1988) Food web dynamics in the ocean .1. Best estimates of flow networks using inverse methods. *Marine Ecology Progress Series,* 42, 269-287.

**Supplementary Table 1 –** Model Solutions (mmol N m^-2^ d^-1^) for each process, model layer (upper euphotic zone (UEZ), deep chlorophyll maximum (DCM), and Twilight zones), during Cycles 1 and 5.

| **Process** | **Model Layer** | **Cycle 1** | **Cycle 5** |
| --- | --- | --- | --- |
| Upwelled NO_3_ | UEZ | 4.37E-5 ± 2.51E-5 | 4.34E-5 ± 2.50E-5 |
| Lateral Input of DON | UEZ | 0.187 ± 0.123 | 0.1 ± 0.098 |
| Lateral Input of PON | UEZ | 0.179 ± 0.137 | 0.943 ± 0.329 |
| Nitrification | UEZ | 0.445 ± 0.177 | 0.792 ± 0.246 |
| N_2_ fixation by PICO | UEZ | 0.09 ± 0.085 | 0.06 ± 0.059 |
| NO_3_ uptake by PICO | UEZ | 0.17 ± 0.139 | 0.363 ± 0.259 |
| NH_4_ uptake by PICO | UEZ | 1.528 ± 0.332 | 3.64 ± 0.391 |
| PICO ==> HNF | UEZ | 0.462 ± 0.27 | 0.958 ± 0.521 |
| PICO ==> MIC | UEZ | 0.273 ± 0.2 | 0.888 ± 0.517 |
| PICO ==> FLAG | UEZ | 2.18E-4 ± 1.74E-4 | 3.69E-4 ± 2.95E-4 |
| PICO ==> APP | UEZ | 2.65E-4 ± 1.70E-4 | 3.97E-4 ± 2.65E-4 |
| DON exudation by TRICHO | UEZ | 0.502 ± 0.185 | 1.336 ± 0.183 |
| TRICHO mortality | UEZ | 0.55 ± 0.262 | 0.881 ± 0.191 |
| N2 fixation by TRICHO | UEZ | 1.90E-3 ± 9.24E-4 | 5.57E-5 ± 3.88E-5 |
| NO3 uptake by TRICHO | UEZ | 1.73E-3 ± 1.21E-3 | 6.51E-5 ± 4.69E-5 |
| NH4 uptake by TRICHO | UEZ | 1.66E-3 ± 1.13E-3 | 6.39E-5 ± 4.62E-5 |
| DON exudation by TRICHO | UEZ | 2.71E-3 ± 1.62E-3 | 9.20E-5 ± 5.79E-5 |
| TRICHO mortality | UEZ | 2.57E-3 ± 1.53E-3 | 9.28E-5 ± 5.78E-5 |
| NO3 uptake by DTM | UEZ | 8.07E-2 ± 4.58E-2 | 8.67E-3 ± 4.99E-3 |
| NH4 uptake by DTM | UEZ | 7.16E-2 ± 4.85E-2 | 7.79E-3 ± 4.92E-3 |
| DTM ==> MIC | UEZ | 3.10E-2 ± 1.35E-2 | 1.82E-3 ± 1.49E-3 |
| DTM ==> HERBnvm | UEZ | 3.31E-3 ± 5.93E-4 | 1.06E-3 ± 9.48E-4 |
| DTM ==> HERBvm | UEZ | 1.33E-2 ± 1.14E-2 | 1.10E-3 ± 1.05E-3 |
| DTM ==> APP | UEZ | 1.97E-4 ± 1.56E-4 | 2.46E-4 ± 2.25E-4 |
| DTM ==> CLAD | UEZ | 1.25E-4 ± 7.91E-5 | 1.37E-4 ± 1.08E-4 |
| DTM ==> nvmCAL | UEZ | 6.57E-3 ± 3.73E-3 | 1.10E-3 ± 1.01E-3 |
| DTM ==> vmCAL | UEZ | 1.42E-2 ± 1.26E-2 | 1.12E-3 ± 1.06E-3 |
| DON exudation by DTM | UEZ | 3.53E-2 ± 1.91E-2 | 3.80E-3 ± 2.00E-3 |
| DTM mortality | UEZ | 2.54E-2 ± 2.04E-2 | 1.15E-3 ± 1.06E-3 |
| NO3 uptake by FLAG | UEZ | 0.193 ± 0.151 | 0.421 ± 0.251 |
| NH4 uptake by FLAG | UEZ | 1.192 ± 0.296 | 0.575 ± 0.301 |
| FLAG ==> MIC | UEZ | 1.001 ± 0.17 | 0.418 ± 0.09 |
| FLAG ==> HERBnvm | UEZ | 0.034 ± 0.027 | 0.087 ± 0.058 |
| FLAG ==> HERBvm | UEZ | 0.034 ± 0.029 | 0.1 ± 0.076 |
| FLAG ==> APP | UEZ | 1.74E-4 ± 1.50E-4 | 2.66E-4 ± 2.24E-4 |
| FLAG ==> CLAD | UEZ | 7.69E-5 ± 6.65E-5 | 1.53E-4 ± 1.20E-4 |
| FLAG ==> nvmCAL | UEZ | 0.063 ± 0.055 | 0.147 ± 0.101 |
| FLAG ==> vmCAL | UEZ | 0.042 ± 0.033 | 0.147 ± 0.084 |
| DON exudation by FLAG | UEZ | 0.391 ± 0.143 | 0.284 ± 0.103 |
| FLAG mortality | UEZ | 0.102 ± 0.066 | 0.106 ± 0.073 |
| HNF ==> MIC | UEZ | 0.137 ± 0.121 | 0.087 ± 0.085 |
| HNF ==> APP | UEZ | 1.63E-4 ± 1.45E-4 | 2.50E-4 ± 2.18E-4 |
| HNF ==> HERBnvm | UEZ | 0.041 ± 0.03 | 0.065 ± 0.054 |
| HNF ==> HERBvm | UEZ | 0.144 ± 0.103 | 0.646 ± 0.371 |
| HNF ==> CLAD | UEZ | 7.28E-5 ± 6.42E-5 | 1.40E-4 ± 1.13E-4 |
| HNF ==> nvmCAL | UEZ | 0.194 ± 0.139 | 0.266 ± 0.18 |
| HNF ==> vmCAL | UEZ | 0.111 ± 0.074 | 0.336 ± 0.237 |
| HNF NH_4_ excretion | UEZ | 0.495 ± 0.127 | 0.802 ± 0.176 |
| HNF DON excretion | UEZ | 0.295 ± 0.094 | 0.454 ± 0.111 |
| HNF egestion | UEZ | 0.625 ± 0.296 | 1.074 ± 0.304 |
| MIC ==> HERBnvm | UEZ | 0.044 ± 0.031 | 0.076 ± 0.058 |
| MIC ==> HERBvm | UEZ | 0.198 ± 0.123 | 0.46 ± 0.283 |
| MIC ==> CLAD | UEZ | 6.71E-5 ± 5.99E-5 | 1.43E-4 ± 1.16E-4 |
| MIC ==> nvmCAL | UEZ | 0.246 ± 0.141 | 0.238 ± 0.158 |
| MIC ==> vmCAL | UEZ | 0.131 ± 0.077 | 0.329 ± 0.214 |
| MIC ==> PREFLEX | UEZ | 3.73E-7 ± 1.57E-7 | 1.15E-8 ± 6.39E-9 |
| MIC ==> POSTFLEX | UEZ | 5.49E-6 ± 8.86E-7 | 1.25E-8 ± 6.61E-9 |
| MIC NH4 excretion | UEZ | 0.527 ± 0.12 | 0.656 ± 0.159 |
| MIC DON excretion | UEZ | 0.319 ± 0.094 | 0.376 ± 0.105 |
| MIC egestion | UEZ | 0.711 ± 0.301 | 0.865 ± 0.27 |
| HERBnvm ==> CHAETO | UEZ | 0.012 ± 0.01 | 0.026 ± 0.02 |
| HERBnvm ==> POECIL | UEZ | 0.012 ± 0.01 | 0.022 ± 0.017 |
| HERBnvm ==> GELPRED | UEZ | 0.012 ± 0.01 | 0.023 ± 0.019 |
| HERBnvm ==> PLANKFISH | UEZ | 0.012 ± 0.01 | 0.018 ± 0.016 |
| HERBnvm NH4 excretion | UEZ | 0.042 ± 0.01 | 0.074 ± 0.018 |
| HERBnvm DON excretion | UEZ | 0.023 ± 0.007 | 0.042 ± 0.012 |
| HERBnvm egestion | UEZ | 0.033 ± 0.012 | 0.073 ± 0.026 |
| APP ==> CHAETO | UEZ | 9.42E-5 ± 8.12E-5 | 1.37E-4 ± 1.18E-4 |
| APP ==> POECIL | UEZ | 9.42E-5 ± 8.04E-5 | 1.35E-4 ± 1.17E-4 |
| APP ==> GELPRED | UEZ | 9.35E-5 ± 7.93E-5 | 1.35E-4 ± 1.16E-4 |
| APP ==> PLANKFISH | UEZ | 9.33E-5 ± 7.94E-5 | 1.35E-4 ± 1.17E-4 |
| APP ==> PREFLEX | UEZ | 8.43E-7 ± 3.06E-7 | 4.87E-8 ± 2.64E-8 |
| APP ==> POSTFLEX | UEZ | 6.77E-6 ± 4.72E-7 | 1.89E-6 ± 1.97E-7 |
| APP NH4 excretion | UEZ | 3.15E-4 ± 9.05E-5 | 5.00E-4 ± 1.50E-4 |
| APP DON excretion | UEZ | 1.68E-4 ± 5.43E-5 | 2.58E-4 ± 9.01E-5 |
| APP egestion | UEZ | 2.41E-4 ± 9.88E-5 | 3.74E-4 ± 1.56E-4 |
| CLAD ==> CHAETO | UEZ | 2.64E-5 ± 2.15E-5 | 4.98E-5 ± 4.16E-5 |
| CLAD ==> POECIL | UEZ | 2.65E-5 ± 2.17E-5 | 5.00E-5 ± 4.10E-5 |
| CLAD ==> GELPRED | UEZ | 2.67E-5 ± 2.17E-5 | 4.87E-5 ± 4.09E-5 |
| CLAD ==> PLANKFISH | UEZ | 2.67E-5 ± 2.16E-5 | 4.93E-5 ± 4.11E-5 |
| CLAD ==> PREFLEX | UEZ | 2.20E-7 ± 1.31E-7 | 8.71E-7 ± 2.44E-7 |
| CLAD ==> POSTFLEX | UEZ | 2.35E-5 ± 2.25E-6 | 2.70E-5 ± 2.76E-6 |
| CLAD NH4 excretion | UEZ | 1.14E-4 ± 2.69E-5 | 2.22E-4 ± 5.41E-5 |
| CLAD DON excretion | UEZ | 6.27E-5 ± 1.75E-5 | 1.20E-4 ± 3.53E-5 |
| CLAD egestion | UEZ | 8.40E-5 ± 3.03E-5 | 1.49E-4 ± 5.60E-5 |
| nvmCAL ==> CHAETO | UEZ | 0.079 ± 0.047 | 0.088 ± 0.067 |
| nvmCAL ==> POECIL | UEZ | 0.039 ± 0.025 | 0.025 ± 0.02 |
| nvmCAL ==> GELPRED | UEZ | 0.068 ± 0.041 | 0.055 ± 0.048 |
| nvmCAL ==> PLANKFISH | UEZ | 0.051 ± 0.037 | 0.051 ± 0.04 |
| nvmCAL ==> PREFLEX | UEZ | 4.75E-6 ± 1.24E-6 | 2.07E-6 ± 3.31E-7 |
| nvmCAL ==> POSTFLEX | UEZ | 6.37E-5 ± 9.63E-7 | 1.35E-5 ± 3.24E-7 |
| nvmCAL NH4 excretion | UEZ | 0.176 ± 0.031 | 0.169 ± 0.04 |
| nvmCAL DON excretion | UEZ | 0.1 ± 0.024 | 0.096 ± 0.027 |
| nvmCAL egestion | UEZ | 0.21 ± 0.059 | 0.223 ± 0.08 |
| CHAETO ==> GELPRED | UEZ | 0.029 ± 0.019 | 0.124 ± 0.074 |
| CHAETO ==> PLANKFISH | UEZ | 0.034 ± 0.02 | 0.057 ± 0.042 |
| CHAETO NH4 excretion | UEZ | 0.061 ± 0.021 | 0.146 ± 0.041 |
| CHAETO DON excretion | UEZ | 0.026 ± 0.015 | 0.046 ± 0.028 |
| CHAETO egestion | UEZ | 0.057 ± 0.025 | 0.21 ± 0.068 |
| POECIL ==> GELPRED | UEZ | 1.03E-2 ± 7.15E-3 | 1.78E-2 ± 7.41E-3 |
| POECIL ==> PLANKFISH | UEZ | 0.033 ± 0.017 | 0.019 ± 0.011 |
| POECIL ==> PREFLEX | UEZ | 1.22E-8 ± 6.56E-9 | 1.24E-8 ± 6.40E-9 |
| POECIL ==> POSTFLEX | UEZ | 2.76E-6 ± 1.84E-6 | 8.93E-7 ± 3.11E-8 |
| POECIL NH4 excretion | UEZ | 0.049 ± 0.014 | 0.037 ± 0.01 |
| POECIL DON excretion | UEZ | 0.017 ± 0.01 | 0.014 ± 0.007 |
| POECIL egestion | UEZ | 0.062 ± 0.022 | 0.037 ± 0.014 |
| GELPRED ==> HTL | UEZ | 0.044 ± 0.016 | 0.134 ± 0.057 |
| GELPRED NH4 excretion | UEZ | 0.049 ± 0.022 | 0.112 ± 0.046 |
| GELPRED DON excretion | UEZ | 0.02 ± 0.004 | 0.046 ± 0.022 |
| GELPRED egestion | UEZ | 0.054 ± 0.025 | 0.216 ± 0.089 |
| PLANKFISH ==> HTL | UEZ | 0.044 ± 0.02 | 0.065 ± 0.032 |
| PLANKFISH NH4 excretion | UEZ | 0.067 ± 0.031 | 0.091 ± 0.039 |
| PLANKFISH DON excretion | UEZ | 0.027 ± 0.012 | 0.041 ± 0.023 |
| PLANKFISH egestion | UEZ | 0.062 ± 0.027 | 0.093 ± 0.044 |
| PREFLEX growth | UEZ | 6.86E-7 ± 4.83E-7 | 3.22E-7 ± 2.34E-7 |
| PREFLEX mortality | UEZ | 6.76E-7 ± 4.75E-7 | 3.52E-7 ± 2.35E-7 |
| PREFLEX NH4 excretion | UEZ | 2.08E-6 ± 7.07E-7 | 1.07E-6 ± 3.10E-7 |
| PREFLEX DON excretion | UEZ | 1.10E-6 ± 4.11E-7 | 5.50E-7 ± 1.86E-7 |
| PREFLEX egestion | UEZ | 1.66E-6 ± 7.28E-7 | 7.18E-7 ± 3.06E-7 |
| POSTFLEX growth | UEZ | 1.14E-5 ± 7.36E-6 | 4.82E-6 ± 3.17E-6 |
| POSTFLEX mortality | UEZ | 1.14E-5 ± 7.43E-6 | 4.90E-6 ± 3.18E-6 |
| POSTFLEX NH4 excretion | UEZ | 3.32E-5 ± 9.10E-6 | 1.39E-5 ± 3.74E-6 |
| POSTFLEX DON excretion | UEZ | 1.77E-5 ± 5.41E-6 | 7.47E-6 ± 2.29E-6 |
| POSTFLEX egestion | UEZ | 2.84E-5 ± 1.05E-5 | 1.22E-5 ± 4.38E-6 |
| BAC ==> FLAG | UEZ | 0.297 ± 0.144 | 0.322 ± 0.165 |
| BAC ==> HNF | UEZ | 0.173 ± 0.137 | 0.294 ± 0.199 |
| BAC ==> MIC | UEZ | 0.125 ± 0.109 | 0.309 ± 0.201 |
| BAC ==> APP | UEZ | 1.25E-4 ± 1.05E-4 | 2.71E-4 ± 2.16E-4 |
| BAC NH4 excretion | UEZ | 1.608 ± 0.223 | 2.329 ± 0.199 |
| DOM uptake by BAC | UEZ | 2.203 ± 0.305 | 3.254 ± 0.277 |
| sDET ==> HNF | UEZ | 1.407 ± 0.585 | 2.479 ± 1.023 |
| sDET ==> MIC | UEZ | 0.609 ± 0.462 | 1.297 ± 0.9 |
| sDET ==> APP | UEZ | 1.82E-4 ± 1.55E-4 | 2.47E-4 ± 2.11E-4 |
| sDET dissolution to DOM | UEZ | 0.15 ± 0.136 | 0.091 ± 0.091 |
| lDET ==> HERBnvm | UEZ | 0.024 ± 0.021 | 0.049 ± 0.044 |
| lDET ==> CLAD | UEZ | 4.97E-5 ± 4.41E-5 | 1.44E-4 ± 1.15E-4 |
| lDET ==> nvmCAL | UEZ | 0.214 ± 0.107 | 0.053 ± 0.039 |
| lDET ==> HERBvm | UEZ | 0.056 ± 0.041 | 0.21 ± 0.171 |
| lDET ==> vmCAL | UEZ | 0.039 ± 0.031 | 0.109 ± 0.106 |
| lDET dissolution to DOM | UEZ | 0.042 ± 0.04 | 0.035 ± 0.036 |
| HERBvm ==> CHAETO | UEZ | 0.056 ± 0.044 | 0.241 ± 0.149 |
| HERBvm ==> POECIL | UEZ | 0.041 ± 0.031 | 0.036 ± 0.027 |
| HERBvm ==> GELPRED | UEZ | 0.031 ± 0.022 | 0.142 ± 0.109 |
| HERBvm ==> PLANKFISH | UEZ | 0.04 ± 0.025 | 0.072 ± 0.061 |
| HERBvm NH4 excretion | UEZ | 0.087 ± 0.035 | 0.278 ± 0.077 |
| HERBvm DOM excretion | UEZ | 0.031 ± 0.024 | 0.13 ± 0.059 |
| HERBvm egestion | UEZ | 0.04 ± 0.026 | 0.269 ± 0.151 |
| HERBvm ==> CHAETO | DCM | 0.034 ± 0.022 | 0.092 ± 0.082 |
| HERBvm ==> POECIL | DCM | 0.03 ± 0.015 | 0.098 ± 0.078 |
| HERBvm ==> GELPRED | DCM | 0.024 ± 0.01 | 0.086 ± 0.077 |
| HERBvm ==> PLANKFISH | DCM | 0.023 ± 0.022 | 0.052 ± 0.048 |
| HERBvm NH4 excretion | DCM | 0.034 ± 0.029 | 0.038 ± 0.036 |
| HERBvm DOM excretion | DCM | 0.043 ± 0.022 | 0.055 ± 0.046 |
| HERBvm egestion | DCM | 0.033 ± 0.025 | 0.098 ± 0.094 |
| HERBvm NH4 excretion | Twilight | 0.03 ± 0.016 | 0.108 ± 0.048 |
| HERBvm DOM excretion | Twilight | 0.017 ± 0.011 | 0.05 ± 0.029 |
| HERBvm egestion | Twilight | 0.027 ± 0.008 | 0.051 ± 0.049 |
| HERBvm Mortality | Twilight | 0.02 ± 0.011 | 0.058 ± 0.056 |
| vmCAL ==> CHAETO | UEZ | 0.06 ± 0.039 | 0.226 ± 0.135 |
| vmCAL ==> POECIL | UEZ | 0.079 ± 0.037 | 0.041 ± 0.028 |
| vmCAL ==> GELPRED | UEZ | 0.017 ± 0.006 | 0.147 ± 0.123 |
| vmCAL ==> PLANKFISH | UEZ | 0.03 ± 0.028 | 0.074 ± 0.064 |
| vmCAL NH4 excretion | UEZ | 0.077 ± 0.034 | 0.321 ± 0.084 |
| vmCAL DOM excretion | UEZ | 0.035 ± 0.019 | 0.157 ± 0.066 |
| vmCAL egestion | UEZ | 0.048 ± 0.028 | 0.432 ± 0.239 |
| vmCAL ==> CHAETO | DCM | 0.006 ± 0.004 | 0.107 ± 0.091 |
| vmCAL ==> POECIL | DCM | 0.008 ± 0.004 | 0.121 ± 0.098 |
| vmCAL ==> GELPRED | DCM | 0.004 ± 0.003 | 0.098 ± 0.089 |
| vmCAL ==> PLANKFISH | DCM | 0.042 ± 0.005 | 0.051 ± 0.046 |
| vmCAL NH4 excretion | DCM | 0.032 ± 0.026 | 0.038 ± 0.037 |
| vmCAL DOM excretion | DCM | 0.025 ± 0.017 | 0.053 ± 0.045 |
| vmCAL egestion | DCM | 0.012 ± 0.007 | 0.122 ± 0.107 |
| vmCAL NH4 excretion | Twilight | 0.027 ± 0.015 | 0.135 ± 0.057 |
| vmCAL DOM excretion | Twilight | 0.016 ± 0.009 | 0.06 ± 0.033 |
| vmCAL egestion | Twilight | 0.032 ± 0.02 | 0.055 ± 0.053 |
| vmCAL Mortality | Twilight | 0.015 ± 0.013 | 0.058 ± 0.059 |
| Sinking DTM | UEZ==>DCM | 2.29E-2 ± 1.33E-2 | 4.93E-3 ± 2.29E-3 |
| Sinking FLAG | UEZ==>DCM | 0.016 ± 0.012 | 0.029 ± 0.015 |
| Sinking lDET | UEZ==>DCM | 1.59E-1 ± 9.17E-2 | 1.00E+0 ± 7.35E-2 |
| Upwelled NO_3_ | DCM | 0.036 ± 0.035 | 0.022 ± 0.022 |
| Lateral Input of DON | DCM | 0.068 ± 0.064 | 0.038 ± 0.036 |
| Lateral Input of PON | DCM | 0.414 ± 0.155 | 0.559 ± 0.272 |
| Nitrification | DCM | 1.131 ± 0.424 | 0.215 ± 0.077 |
| N_2_ fixation by PICO | DCM | 0.027 ± 0.027 | 0.019 ± 0.019 |
| NO_3_ uptake by PICO | DCM | 0.598 ± 0.413 | 0.107 ± 0.077 |
| NH_4_ uptake by PICO | DCM | 0.94 ± 0.428 | 1.682 ± 0.177 |
| PICO ==> HNF | DCM | 0.242 ± 0.117 | 0.256 ± 0.117 |
| PICO ==> MIC | DCM | 0.085 ± 0.074 | 0.082 ± 0.072 |
| PICO ==> FLAG | DCM | 0.114 ± 0.088 | 0.088 ± 0.069 |
| PICO ==> APP | DCM | 0.321 ± 0.125 | 0.528 ± 0.161 |
| DON exudation by TRICHO | DCM | 0.583 ± 0.112 | 0.685 ± 0.099 |
| TRICHO mortality | DCM | 0.221 ± 0.071 | 0.168 ± 0.138 |
| N2 fixation by TRICHO | DCM | 4.12E-5 ± 2.85E-5 | 6.42E-6 ± 4.53E-6 |
| NO3 uptake by TRICHO | DCM | 5.30E-5 ± 3.74E-5 | 7.51E-6 ± 5.43E-6 |
| NH4 uptake by TRICHO | DCM | 5.04E-5 ± 3.63E-5 | 7.64E-6 ± 5.47E-6 |
| DON exudation by TRICHO | DCM | 8.10E-5 ± 4.58E-5 | 1.05E-5 ± 6.67E-6 |
| TRICHO mortality | DCM | 6.36E-5 ± 4.05E-5 | 1.10E-5 ± 6.74E-6 |
| NO3 uptake by DTM | DCM | 3.90E-2 ± 2.52E-2 | 4.24E-3 ± 2.64E-3 |
| NH4 uptake by DTM | DCM | 3.80E-2 ± 2.48E-2 | 4.16E-3 ± 2.70E-3 |
| DTM ==> MIC | DCM | 6.75E-3 ± 4.36E-3 | 3.05E-3 ± 8.71E-4 |
| DTM ==> HERBnvm | DCM | 9.72E-3 ± 8.92E-3 | 1.05E-3 ± 1.00E-3 |
| DTM ==> HERBvm | DCM | 9.41E-3 ± 8.83E-3 | 1.04E-3 ± 9.93E-4 |
| DTM ==> APP | DCM | 9.01E-3 ± 8.38E-3 | 1.03E-3 ± 9.91E-4 |
| DTM ==> CLAD | DCM | 9.95E-3 ± 9.10E-3 | 1.03E-3 ± 9.83E-4 |
| DTM ==> nvmCAL | DCM | 1.00E-2 ± 9.36E-3 | 1.03E-3 ± 9.86E-4 |
| DTM ==> vmCAL | DCM | 8.71E-3 ± 8.37E-3 | 1.02E-3 ± 9.91E-4 |
| DON exudation by DTM | DCM | 1.85E-2 ± 1.00E-2 | 1.98E-3 ± 1.07E-3 |
| DTM mortality | DCM | 1.07E-2 ± 5.78E-3 | 1.06E-3 ± 9.44E-4 |
| NO3 uptake by FLAG | DCM | 0.53 ± 0.319 | 0.126 ± 0.081 |
| NH4 uptake by FLAG | DCM | 0.596 ± 0.352 | 0.363 ± 0.148 |
| FLAG ==> MIC | DCM | 0.589 ± 0.128 | 0.039 ± 0.019 |
| FLAG ==> HERBnvm | DCM | 0.079 ± 0.063 | 0.079 ± 0.061 |
| FLAG ==> HERBvm | DCM | 0.045 ± 0.041 | 0.075 ± 0.065 |
| FLAG ==> APP | DCM | 0.054 ± 0.05 | 0.043 ± 0.04 |
| FLAG ==> CLAD | DCM | 0.092 ± 0.071 | 0.087 ± 0.067 |
| FLAG ==> nvmCAL | DCM | 0.097 ± 0.073 | 0.094 ± 0.07 |
| FLAG ==> vmCAL | DCM | 0.044 ± 0.04 | 0.078 ± 0.068 |
| DON exudation by FLAG | DCM | 0.35 ± 0.093 | 0.142 ± 0.055 |
| FLAG mortality | DCM | 0.127 ± 0.068 | 0.078 ± 0.047 |
| HNF ==> MIC | DCM | 0.045 ± 0.043 | 0.034 ± 0.032 |
| HNF ==> APP | DCM | 0.043 ± 0.041 | 0.031 ± 0.03 |
| HNF ==> HERBnvm | DCM | 0.069 ± 0.058 | 0.052 ± 0.043 |
| HNF ==> HERBvm | DCM | 0.028 ± 0.029 | 0.106 ± 0.076 |
| HNF ==> CLAD | DCM | 0.083 ± 0.066 | 0.043 ± 0.039 |
| HNF ==> nvmCAL | DCM | 0.07 ± 0.058 | 0.047 ± 0.042 |
| HNF ==> vmCAL | DCM | 0.039 ± 0.035 | 0.053 ± 0.054 |
| HNF NH_4_ excretion | DCM | 0.244 ± 0.069 | 0.224 ± 0.066 |
| HNF DON excretion | DCM | 0.142 ± 0.045 | 0.128 ± 0.04 |
| HNF egestion | DCM | 0.306 ± 0.128 | 0.295 ± 0.113 |
| MIC ==> HERBnvm | DCM | 0.105 ± 0.076 | 0.055 ± 0.047 |
| MIC ==> HERBvm | DCM | 0.063 ± 0.051 | 0.056 ± 0.053 |
| MIC ==> CLAD | DCM | 0.108 ± 0.081 | 0.045 ± 0.04 |
| MIC ==> nvmCAL | DCM | 0.101 ± 0.075 | 0.047 ± 0.041 |
| MIC ==> vmCAL | DCM | 0.071 ± 0.044 | 0.05 ± 0.045 |
| MIC NH4 excretion | DCM | 0.287 ± 0.064 | 0.168 ± 0.052 |
| MIC DON excretion | DCM | 0.167 ± 0.046 | 0.098 ± 0.034 |
| MIC egestion | DCM | 0.373 ± 0.122 | 0.216 ± 0.093 |
| HERBnvm ==> CHAETO | DCM | 0.045 ± 0.027 | 0.023 ± 0.019 |
| HERBnvm ==> POECIL | DCM | 0.046 ± 0.039 | 0.028 ± 0.024 |
| HERBnvm ==> GELPRED | DCM | 0.037 ± 0.034 | 0.025 ± 0.023 |
| HERBnvm ==> PLANKFISH | DCM | 0.032 ± 0.029 | 0.024 ± 0.022 |
| HERBnvm NH4 excretion | DCM | 0.119 ± 0.037 | 0.077 ± 0.026 |
| HERBnvm DON excretion | DCM | 0.135 ± 0.071 | 0.088 ± 0.044 |
| HERBnvm egestion | DCM | 0.069 ± 0.025 | 0.045 ± 0.017 |
| APP ==> CHAETO | DCM | 0.078 ± 0.058 | 0.087 ± 0.067 |
| APP ==> POECIL | DCM | 0.08 ± 0.059 | 0.087 ± 0.066 |
| APP ==> GELPRED | DCM | 0.055 ± 0.047 | 0.069 ± 0.059 |
| APP ==> PLANKFISH | DCM | 0.049 ± 0.044 | 0.055 ± 0.048 |
| APP NH4 excretion | DCM | 0.142 ± 0.048 | 0.111 ± 0.036 |
| APP DON excretion | DCM | 0.073 ± 0.032 | 0.059 ± 0.025 |
| APP egestion | DCM | 0.273 ± 0.092 | 0.381 ± 0.107 |
| CLAD ==> CHAETO | DCM | 0.041 ± 0.033 | 0.028 ± 0.025 |
| CLAD ==> POECIL | DCM | 0.039 ± 0.032 | 0.027 ± 0.022 |
| CLAD ==> GELPRED | DCM | 0.029 ± 0.027 | 0.026 ± 0.024 |
| CLAD ==> PLANKFISH | DCM | 0.022 ± 0.019 | 0.027 ± 0.023 |
| CLAD NH4 excretion | DCM | 0.099 ± 0.033 | 0.085 ± 0.029 |
| CLAD DON excretion | DCM | 0.048 ± 0.022 | 0.043 ± 0.02 |
| CLAD egestion | DCM | 0.114 ± 0.058 | 0.104 ± 0.052 |
| nvmCAL ==> CHAETO | DCM | 0.044 ± 0.039 | 0.028 ± 0.025 |
| nvmCAL ==> POECIL | DCM | 0.047 ± 0.04 | 0.029 ± 0.027 |
| nvmCAL ==> GELPRED | DCM | 0.037 ± 0.033 | 0.025 ± 0.023 |
| nvmCAL ==> PLANKFISH | DCM | 0.032 ± 0.03 | 0.024 ± 0.022 |
| nvmCAL NH4 excretion | DCM | 0.118 ± 0.039 | 0.083 ± 0.029 |
| nvmCAL DON excretion | DCM | 0.059 ± 0.025 | 0.041 ± 0.019 |
| nvmCAL egestion | DCM | 0.14 ± 0.072 | 0.101 ± 0.052 |
| CHAETO ==> GELPRED | DCM | 0.04 ± 0.027 | 0.065 ± 0.047 |
| CHAETO ==> PLANKFISH | DCM | 0.034 ± 0.025 | 0.052 ± 0.041 |
| CHAETO NH4 excretion | DCM | 0.065 ± 0.024 | 0.064 ± 0.025 |
| CHAETO DON excretion | DCM | 0.036 ± 0.016 | 0.034 ± 0.016 |
| CHAETO egestion | DCM | 0.074 ± 0.035 | 0.149 ± 0.068 |
| POECIL ==> GELPRED | DCM | 0.04 ± 0.027 | 0.074 ± 0.051 |
| POECIL ==> PLANKFISH | DCM | 0.031 ± 0.024 | 0.056 ± 0.043 |
| POECIL NH4 excretion | DCM | 0.065 ± 0.025 | 0.062 ± 0.024 |
| POECIL DON excretion | DCM | 0.036 ± 0.016 | 0.033 ± 0.015 |
| POECIL egestion | DCM | 0.078 ± 0.036 | 0.164 ± 0.069 |
| GELPRED ==> HTL | DCM | 0.067 ± 0.026 | 0.14 ± 0.059 |
| GELPRED NH4 excretion | DCM | 0.07 ± 0.024 | 0.085 ± 0.028 |
| GELPRED DON excretion | DCM | 0.037 ± 0.016 | 0.044 ± 0.02 |
| GELPRED egestion | DCM | 0.091 ± 0.039 | 0.2 ± 0.076 |
| PLANKFISH ==> HTL | DCM | 0.049 ± 0.024 | 0.072 ± 0.033 |
| PLANKFISH NH4 excretion | DCM | 0.08 ± 0.029 | 0.086 ± 0.03 |
| PLANKFISH DON excretion | DCM | 0.041 ± 0.019 | 0.045 ± 0.02 |
| PLANKFISH egestion | DCM | 0.095 ± 0.041 | 0.139 ± 0.054 |
| BAC ==> FLAG | DCM | 0.237 ± 0.121 | 0.17 ± 0.098 |
| BAC ==> HNF | DCM | 0.102 ± 0.095 | 0.101 ± 0.08 |
| BAC ==> MIC | DCM | 0.082 ± 0.073 | 0.098 ± 0.076 |
| BAC ==> APP | DCM | 0.12 ± 0.088 | 0.092 ± 0.071 |
| BAC NH4 excretion | DCM | 1.35 ± 0.125 | 1.142 ± 0.102 |
| DOM uptake by BAC | DCM | 1.89 ± 0.175 | 1.603 ± 0.144 |
| sDET ==> HNF | DCM | 0.726 ± 0.359 | 0.656 ± 0.353 |
| sDET ==> MIC | DCM | 0.468 ± 0.283 | 0.479 ± 0.248 |
| sDET ==> APP | DCM | 0.203 ± 0.152 | 0.152 ± 0.131 |
| sDET dissolution to DOM | DCM | 0.044 ± 0.044 | 0.031 ± 0.03 |
| lDET ==> HERBnvm | DCM | 0.22 ± 0.155 | 0.122 ± 0.097 |
| lDET ==> CLAD | DCM | 0.1 ± 0.096 | 0.163 ± 0.117 |
| lDET ==> nvmCAL | DCM | 0.199 ± 0.148 | 0.143 ± 0.112 |
| lDET ==> HERBvm | DCM | 0.049 ± 0.038 | 0.3 ± 0.224 |
| lDET ==> vmCAL | DCM | 0.066 ± 0.049 | 1.189 ± 0.384 |
| lDET dissolution to DOM | DCM | 0.049 ± 0.045 | 0.028 ± 0.028 |
| Sinking DTM | DCM==>Twilight | 0.007 ± 0.006 | 0.001 ± 0.001 |
| Sinking FLAG | DCM==>Twilight | 0.016 ± 0.01 | 0.057 ± 0.031 |
| Sinking lDET | DCM==>Twilight | 0.436 ± 0.026 | 0.465 ± 0.178 |
